# Mass Spectrometry Imaging

**DOI:** 10.1021/acs.analchem.4c05249

**Published:** 2025-07-17

**Authors:** Aljoscha Körber, Ian G. M. Anthony, Ron M. A. Heeren

**Affiliations:** † The Maastricht MultiModal Molecular Imaging Institute (M4i), Division of Imaging Mass Spectrometry, 5211 Maastricht University, Universiteitssingel 50, 6229 ER Maastricht, the Netherlands

## Abstract

Mass spectrometry imaging (MSI) maps the spatial distributions of chemicals on chemically complex surfaces. MSI offers unrivaled sensitivity and information density with each pixel comprising a mass spectrum. Over the past three decades, numerous technological developments have enabled MSI to evolve into a mainstream technique for untargeted molecular and elemental imaging with wide-spread applications ranging from material analysis to life sciences and clinical diagnostics. Here, we review the field of MSI with a focus on key technological advancements. We examine different image acquisition modes and the most popular ionization methods in MSI, including matrix-assisted laser desorption/ionization (MALDI), laser ablation inductively coupled plasma (LA-ICP), laser ablation electrospray ionization (LAESI), secondary ion mass spectrometry (SIMS), and desorption electrospray ionization (DESI). For each method, we discuss figures of merit, such as spatial resolving power and sensitivity, the ionization mechanism, sample preparation, advantages, and disadvantages, including ways to overcome them wherever applicable. We subsequently discuss more aspects of MSI instrumentation, such as commonly used mass analyzers, tandem mass spectrometry, ion mobility, and advancements in imaging throughput. Based on these technological developments, targeted MSI strategies are explained, including imaging mass cytometry (IMC), multiplexed ion beam imaging (MIBI), and stable isotope labeling (SIL), as well as approaches for multimodal imaging. Last, we present selected application examples of MSI in cancer research, single cell analysis, and drug distribution studies. We target this review to provide researchers with an interest in recent developments in MSI with a concise technological understanding of the different main approaches to MSI.

## Graphical abstract

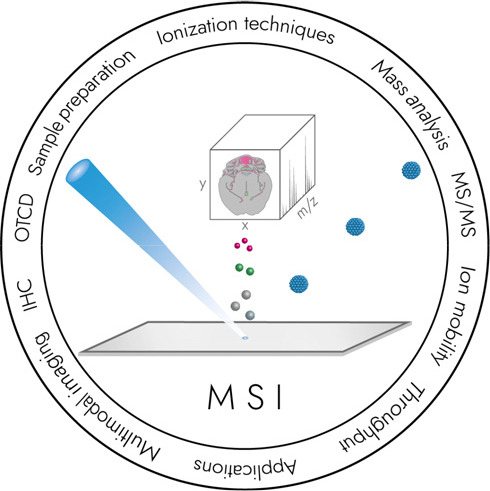

## Introduction

Observation of features and objects smaller than the human eye can resolve has been an enabler for scientific discoveries ranging from material sciences and structural biology to forensics and medical diagnostics. The information obtained from imaging techniques has been so valuable that a large part of modern research would seem inconceivable without them. However, despite impressive spatial resolving capabilities, the majority of imaging techniques lack chemical specificity as their observables often cannot be attributed to single identified molecules, especially on complex surfaces. For instance, light and scanning electron microscopy can visualize cell organelles, but do not provide any information on, for example, the cellular proteome or metabolome. Labeled approaches, like immunofluorescence microscopy, can yield insights on labelled molecules, though it is difficult to monitor more than 3–5 molecules simultaneously,^1,2^ due to spectral overlap, extensive sample preparation, and the lack of suited labels.^3^ Considering that each cell consists of millions of different molecular species, it is not possible to image even a moderate fraction of the proteome, metabolome, lipidome, etc. with labeled imaging approaches alone. The lack of chemical specificity in imaging is disadvantageous as almost every disease is linked to biochemical change. Thus, better observations and measurements of the biochemistry of cells will help in diagnosing, understanding, and combatting disease. Bulk analysis methods, such as high-performance liquid chromatography mass spectrometry (HPLC-MS), contribute to our understanding, but do not provide spatial information on local chemistry, such as the microenvironment of tumor cells. Furthermore, biomolecules often possess different functions at different locations and concentrations in tissues and cells. Thus, techniques are needed that allow detecting, identifying, quantifying, *and* localizing thousands of different molecules of varying abundances in complex samples.

One promising technique to bridge the gap between high molecular and high spatial information is Mass Spectrometry Imaging (MSI). MSI allows the measurement and visualization of the spatial distributions of atoms and molecules on a surface as well as their subsequent identification by means of mass spectrometry.^4,5^

In comparison to other imaging techniques, MSI offers unrivaled molecular information density, high sensitivity, and is applicable to chemically complex surfaces. Furthermore, MSI is untargeted and requires neither labeling nor other extensive sample preparation — yet can be applied in a targeted manner to reveal valuable and easily-understandable information about a surface. MSI measurements can yield large amounts of data, which, although sometimes challenging to store and analyze, allow for extensive exploratory studies. Although the spatial resolving power and throughput of MSI are currently poorer than other imaging methods, recent technological advancements are decreasing these differences.^6–8^

Consequently, MSI has entered the mainstream of common analysis methods, with a wide range of applications and more than 1,000 publications per year according to PubMed. The progress in instrumentation and data handling over the last decade as well as the growing demand for single cell and tissue analysis is expected to boost the popularity of MSI further.^9–11^

Here, we review the field of MSI with a focus on the fundamental principles as well as on the technological advancements that have enabled MSI to evolve into a mainstream technique in the last 20 years.^4^ We focus less on applications and also do not cover less commonly used imaging techniques, such as those based on continuous liquid extraction, like “nano-DESI”,^12^ approaches primarily used for sampling instead of imaging,^13,14^ or MSI data analysis strategies, as this topic is comprehensively reviewed in the data sciences domain.^15^ We begin by defining different image acquisition modes of MSI. Then, we evaluate the most common laser- and charged particle-based MSI ionization methods, each with an introduction, mechanism discussion, sample preparation protocols, if applicable, and discussion of the major challenges each method faces, including ways to overcome them. Next, we review mass spectrometer instrumentation including different mass analyzers, ion mobility spectrometry (IMS), tandem MS techniques, and imaging throughput. We then present targeted MSI strategies and subsequently review the application and potential benefits of MSI combined with other techniques, like optical and electron microscopy, in multimodal imaging approaches.^5^ At the end of the review, we highlight some selected application examples of MSI.

## Modes and Concepts of MSI

### Microprobe and Microscope Modes

There are two methods to perform MSI, microprobe- and microscope-mode. The latter is also known as “stigmatic ion imaging”, “direct imaging”,^16^ and “mass microscopy”. Almost all modern-day MSI is performed in microprobe-mode (Figure 1a), in which the sample is scanned pixel-by-pixel with a focused ionizing beam or surface probe. A variation of microprobe-mode MSI is using a line scan, where the sample is moving continuously while mass spectra are acquired. Retrospectively, the spectra are re-binned into individual pixels. Microscope-mode, henceforth mass microscopy for easier reading, makes use of a *defocused* ionizing beam to extract a, comparatively large, ion image from the sample, which is preserved throughout the mass analyzer, and projected onto a spatially-sensitive detector (Figure 1b). In a pixelated, spatially-sensitive detector with a time-of-flight (TOF) based mass microscope, every pixel of the detector corresponds to a single mass spectrum, which allows tens of thousands of mass spectra to be acquired in parallel.^4^ Areas larger than the focus size can be acquired by moving the sample (see imaging throughput).^8^

**Figure 1:**
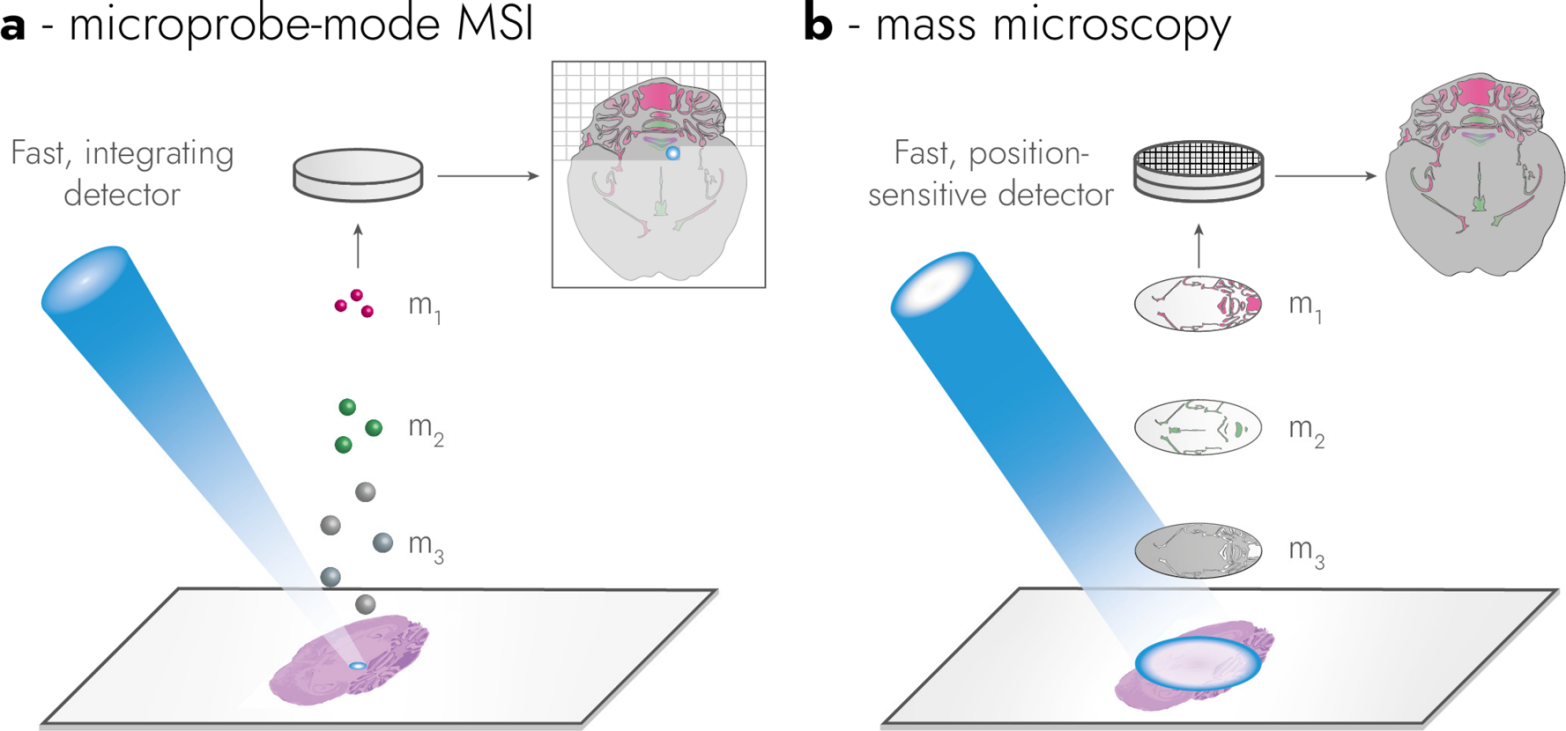
Operating principles of TOF-based microprobe-mode MSI and mass microscopy. In microprobe-mode MSI (a) a focused ionizing beam scans the sample and sequentially acquires mass spectra for every pixel. In mass microscopy (b) a defocused ionizing beam irradiates a large part of the sample causing the formation of an ion image on the surface. This image is then extracted into the gas phase, preserved during mass analysis, and magnified onto a fast, spatially sensitive detector. The ionizing beams are depicted in blue, and ions of different mass-to-charge are for illustrative purposes annotated m_1_, m_2_, and m_3_, respectively.

The achievable spatial resolving power and spatial resolution are crucial figures of merit in every imaging technique. Both terms are often used synonymously but their meanings differ. In this review, we define spatial resolution as the chosen pixel size of an MSI acquisition,^4^ whereas spatial resolving power is a fixed, instrument-specific parameter defined as either the smallest distance between two points, or the 20%-80% rising edge of a sharp, defined image feature, e.g. bars of a TEM grid, that can reproducibly be resolved with sufficient contrast, sampling, *and* sensitivity.^4^ Sensitivity in particular is a limiting factor in high spatial resolution microprobe-mode MSI because probing smaller volumes implies decreasing the amount of material, which can be desorbed and ionized. Next to sensitivity and techniques such as expansion microscopy applied to MSI,^17,18^ spatial resolving power in microprobe-mode is ultimately limited by the smallest achievable two-dimensional spot size of the ionizing beam, delocalization that might occur during preparation or transfer, and eventual sample damages inflicted to the surroundings of the sampling spot. In mass microscopy, spatial resolving power is theoretically ion-diffraction limited.^19^ In practice, the quality of ion optics and detector, ion energy spread caused by the ionization event, and space-charge effects limit lateral resolving power.^20^ Values of ∼110 nm, 1 μm, 3.4 μm have been reported using secondary ion mass spectrometry (SIMS) on a magnetic sector,^19^ pulsed time-of-flight (TOF)-SIMS and laser desorption ionization (LDI),^21,22^ and matrix-assisted laser desorption/ionization (MALDI), respectively.^23^

Although achievable spatial resolving power of an instrument is constant, spatial resolution can be increased by decreasing the step size in microprobe mode or by increasing the magnification in mass microscopy. There are two particularities in cases of oversampling, which occurs when the pixel size becomes smaller than the lateral resolving power. First, if a microprobe-mode MSI technique depletes the sample or the matrix necessary to observe ion signal, the effective spatial resolving power can be increased by moving the sample by a distance smaller than the focus size of the ionizing beam. In practice, this gain is limited by elongated acquisition times and a drop in sensitivity as smaller volumes are sampled.^24,25^ Second, in mass microscopy the sample can be moved constantly while continuously recording incoming ion images. These images may be fused into an overview image, which has arbitrarily small oversampling amounts. In this case, image resolution must be chosen during data viewing and can in principle use pixels of any size. In practice, the effective pixel size is limited by Nyquist-Shannon sampling criteria.^8^

Microprobe-mode and mass microscopy have complementary advantages and disadvantages: First, due to parallel acquisition of mass spectra, mass microscopy allows for orders of magnitude higher throughput than microprobe-mode (see chapter on imaging throughput).^8^ Furthermore, mass microscopy does not require a focused ionizing beam. However, microscope-mode MSI can only be done under high vacuum (HV) with stigmatic time-of-flight (TOF) or magnetic sector mass analyzers.^16,19,21^ Microprobe-mode MSI can, depending on ionization technique, be performed at higher or atmospheric pressures, which allows studying volatile and labile molecules. Furthermore, microprobe-mode MSI can be coupled to essentially any type of mass analyzer, be used with ion mobility techniques, and is conducive to two-step ionization techniques. These reasons and the lack of spatial detectors with sufficient time resolution have made microprobe-mode become the dominant acquisition method in MSI. Recent developments of novel fast detectors and the need for high throughput, high-resolution MSI however,^8,26–28^ have led to renewed interest in mass microscopy.

### Three-Dimensional MSI

MSI can be expanded to a third spatial dimension by the acquisition of topology-correlated 2D images or by the construction of a three-dimensional (3D) image from several 2D images of different layers of the sample.^29^

First topology-correlated imaging is important for the high-resolution analysis of rough surfaces in microprobe-mode as reducing the lateral focal size also reduces focus depth.^29^ Objects out of focus will not be ionized efficiently. Notably, this is less of an issue in mass microscopy, which can image objects varying in height by more than 100 μm.^8^ To correct for sample topology, surface height variations can be mapped with a confocal chromatic or a laser triangulation sensor,^30,31^ allowing for automated adjustment of sample height.

Second, 3D MSI is either performed by sectioning a sample into thin slices and acquiring 2D images of the separated slice surfaces or by repeatedly removing a layer of the sample and imaging the freshly-exposed material.^29^ Slicing is available to all MSI methods but has several challenges: First, slicing the sample and putting the resulting tissue sections onto an imaging surface presents an extra working step. This working step is, especially when performed manually, prone to causing artefacts, like tears and folds in the slices, or even the loss of some slices.^32,33^ Second, all slices should be measured in the shortest time interval possible to minimize variations in instrument performance.^32^ This is difficult to achieve at high spatial resolution with state-of-the-art microprobe-mode MSI instruments due to their correspondingly limited throughput. Third, for the creation of an accurate 3D model, the 2D images must be co-registered with a precision greater than the lateral resolution of the experiment.^32,34^

Removing layers of sample material is only available to MSI methods that possess ion sputtering or laser ablation capabilities. Ion sputtering offers far superior depth resolutions compared to successive slicing.^35^

## Laser-Based Ionization Methods

### Matrix-Assisted Laser Desorption/Ionization (MALDI)

#### Theory and Imaging

Matrix-Assisted Laser Desorption/Ionization (MALDI) is the most widely used MSI ionization method. MALDI is a sensitive ionization technique that yields primarily intact single charged ions over a broad mass range, from metabolites to proteins. Commercial instruments reach spatial resolving powers down to 5 μm,^36^ while ahead of the state-of-the-art instruments have achieved spatial resolving powers below 1 μm.^6^

MALDI MSI is predominantly performed with ultraviolet (UV) lasers, while infrared (IR) MALDI MSI suffers from inferior sensitivity and spatial resolving power, and will thus be ignored throughout this review, unless specified otherwise. In MALDI, a matrix chemical, usually an easy-to-desorb UV-absorbing acid or base, is applied to the sample surface, where it locally co-crystallizes with analyte molecules. A laser then irradiates the sample. The matrix molecules absorb the light and desorb in a collective motion together with the entrained analytes. Without matrix, laser desorption ionization (LDI) is limited to low molecular weights, and while we refrain from explicitly featuring LDI here, some points discussed in this section are equally applicable to LDI.

The mechanism by which the matrix enhances ionization is debated. Two main pathway models are generally accepted: the ‘lucky survivors’ model and the MALDI plume proton transfer model (Figure 2).^37–41^

**Figure 2:**
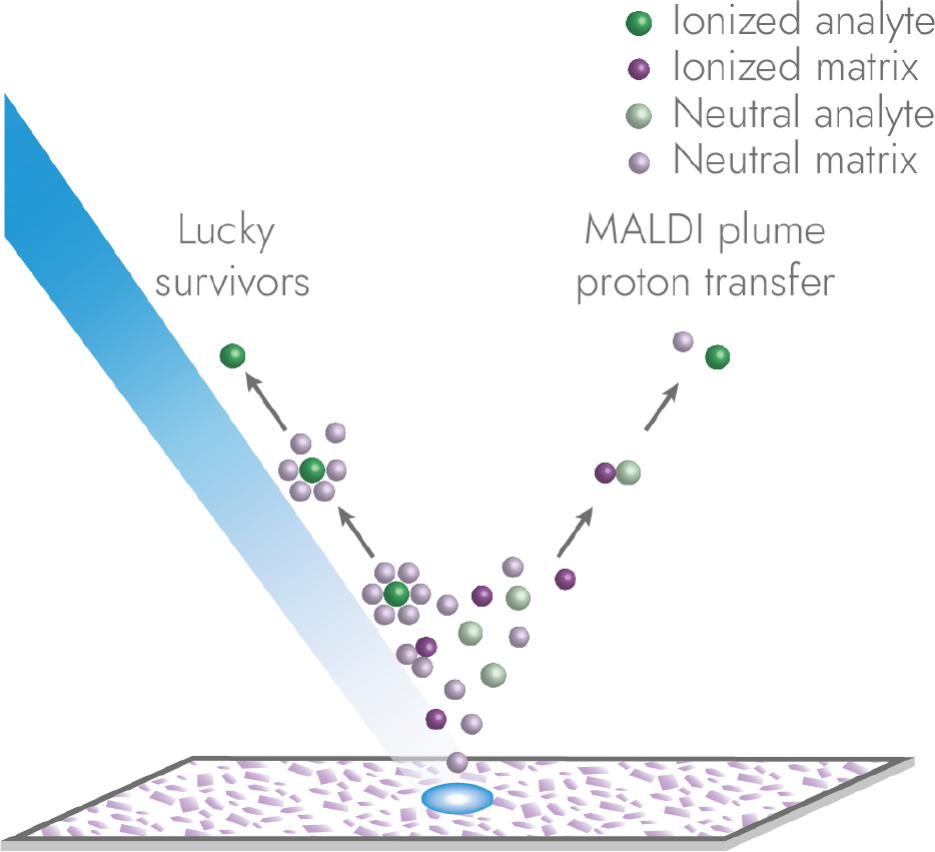
Ionization pathways in MALDI. A laser irradiates a sample surface coated with matrix crystals causing the collective desorption of matrix and analytes into the gas phase. In the ‘lucky survivors’ model ionization occurs on the surface, and the laser merely causes desorption of charged clusters, from which analyte ions can be released and detected. In the MALDI plume proton transfer model , analyte molecules undergo ionization via gas phase collisions with other ionized particles, for instance matrix molecules.

The lucky survivors model assumes that the analytes and matrix molecules already exist in charged states in the matrix layer. The laser irradiation enables separation of ions from their ionic counter parts, leading to ablated clusters with a net charge. These clusters dissociate in the gas phase but preserve their charge in form of a proton, sodium or another ion adduct, which is eventually transferred from the matrix to the analytes. If the charged clusters or ions are not neutralized by colliding with other particles, they can be detected as ‘lucky survivors’. In contrast, the MALDI plume proton transfer model assumes that the ionization of the analytes takes place after desorption via gas phase collision and proton transfer with photoionized matrix molecules or other ions present in the desorption plume. There is experimental evidence for both pathways.^38,39^ To those interested, we recommend articles by Michael Karas and Richard Knochenmuss.^37,38,40^

Three aspects of the mechanism of MALDI are especially important for its application to imaging: ionization efficiency, ion suppression, and the ‘spot size effect’. First, the ionization efficiency, viz. the ratio of ions to desorbed neutrals, is typically in the range of 0.1 to 0.01%.^42,43^ This low efficiency is a limiting factor for high spatial resolution imaging. Second, abundant, easily ionizable or ionized compounds of an opposite charge can suppress the ionization of analyte molecules.^44^ Thus, the ion yield of every molecule depends on its chemical environment. In the literature, this ion suppression is often also referred to as “matrix effects”.^4^ Ion suppression can impair the detection of classes of molecules, and compensating for ion suppression effects can require chemical derivatization of the sample or a second ionization step, like MALDI-2. Third, at laser spot sizes of 20 μm and smaller, the energy threshold required for MALDI increases, while the ion yield decreases.^42,45,46^ This ‘spot size effect’ is important for MSI as it additionally lowers the sensitivity of MALDI imaging at high spatial resolutions. Some evidence suggests that the need for larger laser fluences with smaller spot sizes leads to a transition between two material ejection regimes.^45,46^ Lower fluences favor desorption of smaller, faster individual molecules whereas higher fluences favor desorption of slower, larger molecular clusters. However, Niehaus et al. observed that the amount of ejected material per laser shot is, at constant laser fluence, approximately the same for different spot sizes.^46^ The decrease in ion yield with smaller spot sizes was therefore attributed to the evolution of smaller MALDI plumes with larger surface-to-volume ratios.^46^ Smaller plumes cool and disperse faster, which might prevent efficient declustering and may even allow molecules to condense and form new clusters, which are then not detected.^46^

#### Sample Preparation

A typical sample preparation workflow in imaging may be seen in Figure 3.

**Figure 3:**
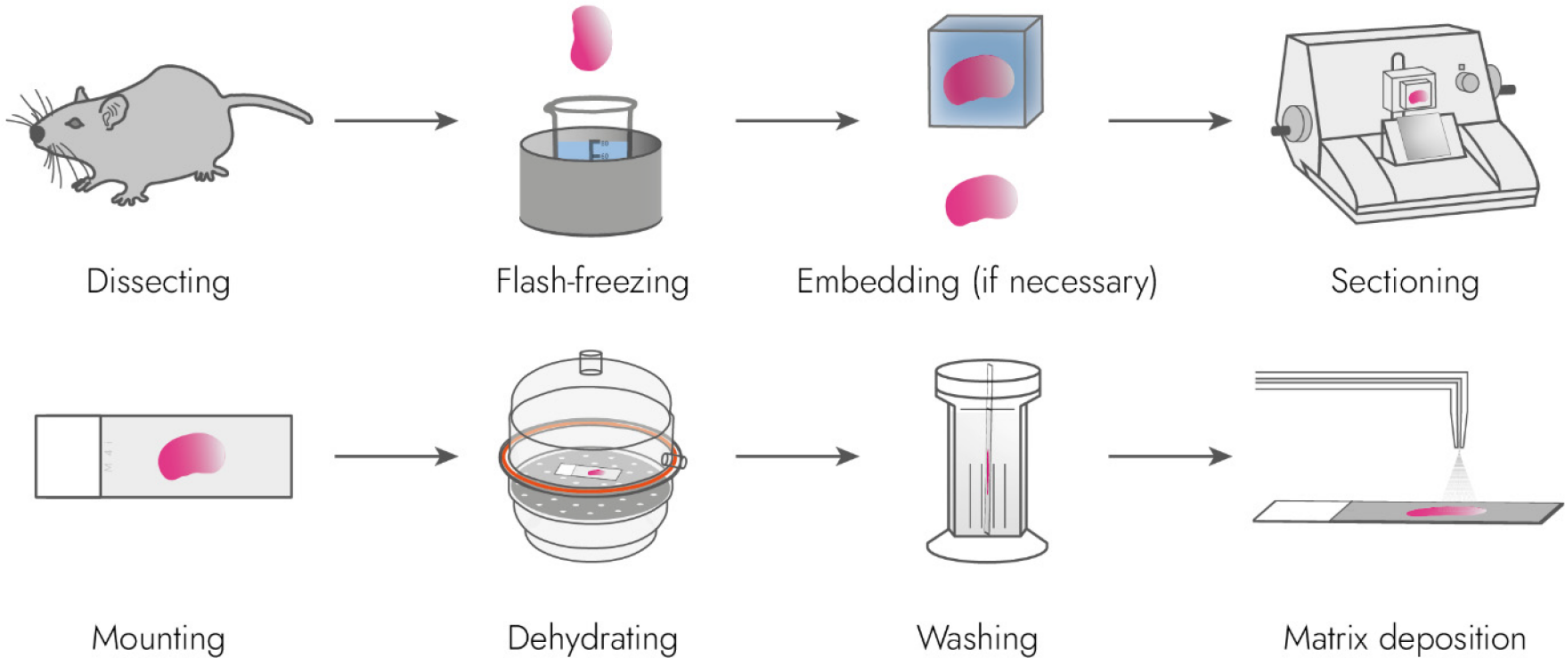
An example sample preparation workflow for MALDI MSI of fresh-frozen tissue. First, a biological sample is retrieved from an organism and flash-frozen via plunging into cryogenic isopentane. Then, if necessary, the tissue is embedded in gelatin to facilitate its sectioning into thin slices, which are then mounted onto microscopy slides. The obtained slides are defrosted under vacuum in a desiccator, which removes volatile compounds, e.g. water, from the tissue and, moreover, prevents water condensation from the atmosphere. Washing tissue slices to remove salts and/or lipids may be required depending on the analytes of interest. Last, matrix is applied to the sample.

The majority of MSI is not performed on chemically fixed samples, but on fresh-frozen tissues to avoid the cross-linking, delocalization or removal of analytes, such as lipids and metabolites, from the sample.^47–49^ Freezing is best performed by plunging recently collected tissue into cryogenically cooled isopentane. Flash-freezing into liquid nitrogen (LN) is also possible in many cases, but due to a lower and uneven cooling rate, can damage the tissue morphology. In this case a more gentle approach for small samples is to wrap the tissue into tin-foil and have it float on the LN surface. Fresh-frozen tissue must be stored at -80° C or below.^50,51^

After freezing, the tissue is sliced into approximately 10 μm thin sections with a cryotome at -20 °C and put onto conductive indium tin oxide (ITO) slides, either via thaw mounting or by use of conductive tape.^52^ If the tissue is difficult to cut, it can be embedded prior to sectioning in gelatin,^53^ carboxymethyl cellulose,^54^ a mixture of hydroxypropyl methyl cellulose (HPMC) and polyvinylpyyrolidone (PVP),^55^ agarose,^56^ or a combination of those, depending on the sample and thereby eventual chemical delocalization and ion suppression. Optimal cutting temperature (OCT) polymer should be avoided for these reasons as it easily ionizes and can lead to polymeric peaks in the spectrum that obscure the analytically relevant peaks.^47,48,53^ After mounting, the slides are either stored or warmed to room temperature and dehydrated in a desiccator. For the analysis of on-tissue digested peptides (e.g. with trypsin) and proteins, lipids and salts are removed by washing the sample for instance with ethanol, xylene, and/or chloroform. Salts and lipids are undesirable as they can cause ion suppression, form adducts, and reduce peptide digestion efficiency.^48,57^ Lipid removal is also recommended for MSI of other molecule classes, e.g. glycans,^58^ and desalting, for instance via washing with ammonium formate, was reported to enhance lipid detection in negative polarity.^59^

Protocols for the analysis of formalin-fixed, paraffin-embedded (FFPE) tissue sections have also been developed due to the widespread use of FFPE in pathology. FFPE tissue sections must be deparaffinized via washing in pure xylene and ethanol.^58,60,61^ Unless metabolites or endogenous peptides are targeted,^60^ this washing step is followed up by a heat-mediated or enzymatic antigen retrieval and proteolytic digestion, typically with trypsin for bottom-up proteomics,^61^ or PNGase F for glycan analysis.^58^ Using these protocols enabled imaging of peptides of decades old samples.^60,62^ However, all results must be interpreted with care due to the age of the samples, their possible chemical degradation, and because the paraffin embedding and removal steps lead to the removal of metabolites and lipids.^50,51^

MALDI MSI can also be combined with expansion microscopy (ExM) to enhance effective spatial resolving power triple to sixfold.^17,63^ In ExM, chemically fixated tissues are linked to a hydrogel, which is subsequently swelled causing tissue expansion.^64^ Chan et al. presented MALDI ExM images with submicrometer pixel size. A large part of all lipids was washed away during the tissue expansion workflow.^17^ Targeted proteomics with photocleavable linkers, resulted in weak ion signals,^17^ but more optimization of the ExM workflow seems possible, for instance by slow thermal instead of proteolytic tissue homogenization.^18^

MALDI matrix selection is crucial as it influences sensitivity, specificity, and spatial resolution in an imaging experiment. A ‘matrix for all needs’ has not been found, and the right choice of a matrix depends on the desired application^65^. The general requirements for a matrix to be suited for MSI are (i) vacuum-compatibility, unless an atmospheric pressure MALDI source is being used, as matrix sublimation and an associated change in matrix to analyte ratio would lead to artefacts in the ion images, (ii) the formation of small uniformly distributed crystals to allow for high spatial resolution and low sampling variance, and (iii) reduced formation of potentially interfering matrix clusters to minimize fragmentation and chemical noise. Further desirable properties are low price, good solubility, and nontoxicity. Commonly used matrices for the analysis of lipids, peptides, metabolites, or other small molecules include 2,5-dihydroxybenzoic acid (DHB) and α-cyano-4-hydroxycinnamic acid (CHCA) in positive polarity,^65^ and 9-aminoacridine (9-AA) and norharmane in negative polarity,^65^ while 1,5-diaminonaphtalene (1,5-DAN), 2,6-dihydroxyacetophenone (2,6-DHA), and dithranol yield ions in both polarities.^65–67^ 2,6-DHA and dithranol sublime quickly under vacuum.^66,67^ Matrices suited for protein imaging in positive polarity are 3,5-Dimethoxy-4-hydroxycinnamic acid (sinapinic acid) and 2,5-Dihydroxyacetophenone (2,5-DHA).^68,69^ A comprehensive list of suited matrices can be found in the literature.^65^

After sectioning and any eventual chemical derivatization, on-tissue digestion, or labeling steps, MALDI matrix must be applied. Although the ‘dried droplet’ approach is common for non-imaging MALDI experiments, it does not yield chemically and morphologically uniform matrix crystals and can lead to variances in signal intensity and other artefacts.^70,71^ Most common instead is to deposit matrix via spraying or sublimation.

Automated sprayers eliminate the risk of human error during matrix deposition and enable the dispersion of much smaller droplets than manual spraying can routinely achieve, thereby reducing the issue of solvent-induced sample diffusion. Commercialized pneumatic sprayers can reproducibly achieve uniform matrix crystals smaller than 10 μm by precisely controlling experimental parameters of the matrix solution and the environment, such as temperature, pressure, and humidity.^72^ However, imaging with a desired spatial resolution below 10 μm benefits from even smaller crystals to avoid ionization of molecules outside the laser focus.

Sublimation can provide smaller crystal sizes than spraying and as such is more attractive for high spatial resolution applications. In sublimation workflows, pure matrix is sublimed and redeposited onto the sample. By performing the redeposition step at -15° C, Xie et al. achieved uniform matrix layers with crystal sizes down to 0.2 μm.^73^ Sublimation does not require solvents that could cause analyte redistribution,^74^ but this absence causes decreased sensitivity compared to spraying because solvents assist analyte migration into matrix crystals.^75,76^ Instead, sample analytes diffuse and enrich in the matrix layer. Diffusion speed, however, is molecule-dependent.^77^ To compensate for the loss of sensitivity compared to spraying, samples prepared by sublimation can undergo a recrystallization step in a humid environment.^75^ Recrystallization can improve sensitivity at the cost of increased analyte diffusion, especially of water-soluble, low molecular weight analytes.^77^ Another strategy worth mentioning is the doping of tissue with a sodium salt prior to sublimation to achieve higher signal intensities of lipid-salt adducts.^78^

#### On-Tissue Chemical Derivatization (OTCD)

On-tissue chemical derivatization (OTCD) is an extra sample preparation step, done prior to or during matrix application, to boost the ionization efficiency of various classes of chemicals, which otherwise would be suppressed, for example neurotransmitters or steroids.^79,80^

Usually the chemical structure of the analyte is altered by adding a charged side group or by tuning the proton affinity of the analyte.^80^ OTCD agents should not only exhibit high conversion and chemical specificity but also work quickly under mild reaction conditions to prevent chemical degradation or analyte delocalization. As chemical derivatization can make mass spectra more complex, it is helpful to avoid peak interferences by increasing the masses of the derivative compounds so that their masses appear in otherwise sparse regions of the mass spectrum. Adding a distinct isotopic pattern, for example a bromine group,^81^ can also facilitate distinguishing between derivatives and unaltered molecules.

OTCD can be simplified if the reagent also serves as the matrix for MALDI. These so-called “reactive matrices” offer the advantages of requiring fewer sample treatment steps, large OTCD reagent to analyte ratios, and uniform application via spraying. For example, neurotransmitters can be imaged at high resolution by targeting their amine groups using fluoropyridinium salts.^82^ Furthermore, some OTCD reagents employ the laser used for UV-MALDI to trigger a photoreaction at the start of the MALDI process.^83,84^ Based on these strategies, there are many different OTCD protocols, on which we refer to a review by Merdas et al. for more information.^80^

#### Ion Source Pressure

Early MALDI sources operated in high vacuum (HV) of less than 10^‑3^ mbar.^42^ In HV, it is unlikely that ions will collide with gas phase molecules or atoms. Therefore, the likelihood of ion losses, collision-induced fragmentation and ion adduct formation is minimal in HV.^85^ Nowadays, most MALDI MSI instruments operate at intermediate or elevated pressures (EP) of 0.5 to 10 mbar.^86–88^ In this pressure regime, collisions between ions and a buffer gas, for example nitrogen, occur and cause collisional cooling, which can prevent the fragmentation of ions with high internal energy or labile chemical moieties and thus lead to higher intact ion signals.^85,86^ Furthermore, EP preserves a dense and reactive MALDI plume for the first few microseconds after laser irradiation allowing for more gas phase collisions and enhanced ionization.^89^ Another practical advantage is that EP sources can be easily coupled to ion mobility spectrometers, which operate at a similar pressure.^87^

Alternatively, MALDI can also be performed at atmospheric pressure (AP). AP MALDI enables the analysis of hydrated samples containing small, volatile molecules, which cannot be detected with EP- or HV MALDI. Moreover, it allows using volatile matrices, which enables longer imaging runs and in some cases can yield enhanced ion signals.^90^ The main disadvantage of AP MALDI is its reduced sensitivity compared to EP- and HV MALDI due to an increased rate of ion losses.^91,92^ The inlet of AP- MALDI mass spectrometers usually is heated to 200-550 °C to improve sensitivity.^92,93^ Another improvement has been the coupling of AP MALDI to MALDI-2 and dielectric barrier discharge ionization (DBDI), which lead to an increase in ion yield for many molecules, especially at lower masses,^92^ and, similarly to MALDI-2 at EP, allowed imaging molecules which previously were not detectable with AP MALDI.^92–94^ However, DBDI also made several ion signals vanish,^92^ and caused increased in-source fragmentation leading to more complicated spectra.^92–94^ Moreover, signal carry-over between pixels has been reported, which might be detrimental to imaging throughput.^93^

#### Spatial Resolving Power

Spatial resolving power in microprobe-mode MALDI is primarily limited by sensitivity (see sections on MALDI-2 and sample preparation) and focus size of the laser beam.

When a collimated laser beam of beam quality factor *M* ^2^ ≥ 1, wavelength *λ*, and beam diameter *D*_*b*_ is focused through an aberration-free lens of focal length *f* onto a sample at an angle of incidence *θ* the resulting elliptical beam diameter *D*_*f*_ at the focal point can be described by the following formula:^95,96^

1 $$\vec{D_{f}}=\left(\begin{array}{c} D_{f,major\;axis}\\ D_{f,minor\;axis}\; \end{array}\right)=M^{2}\cdot \frac{4\lambda f}{\pi D_{b}}\cdot \left(\begin{array}{c} 1/\cos\theta \\ 1 \end{array}\right)$$

Thus, smaller laser spots can be achieved in five ways: (i) reducing the beam quality factor *M* ^2^ towards a Gaussian beam (*M* ^2^ → 1), (ii) using a laser with shorter wavelength *λ*, (iii) expanding the laser beam diameter *D*_*b*_ prior to focusing, (iv) reducing the focal length *f* of the employed objective, and (v) by reducing the angle of incidence *θ*.

Beam quality factor (i, *M* ^2^) is a property of the used laser. Most state-of-the art MALDI instruments use solid-state lasers with small beam quality factors.^97–101^ Older instruments were equipped with nitrogen lasers with poorer beam quality factor. In this case the beam quality factor can be reduced at the expense of energy losses via focusing the laser beam through a pinhole to filtrate off higher-order modes of the laser and achieve a nearly-Gaussian beam (*M* ^2^ → 1).^102^ However, Gaussian beam profiles are not always suited best for MSI due to their beam shape.^99^ On the edges of a Gaussian profile, the laser fluence might be high enough to desorb but too low to efficiently ionize, while in the center of a Gaussian beam the fluence might be too high causing fragmentation and cluster formation. Furthermore, for optimal sample usage, it is better to use flat top square beam profiles.^45^ Consequently, the beam quality factor is sometimes increased for medium spatial resolution MSI by using a beam homogenizer,^45^ or two consecutive coarse diffraction gratings to generate a lattice beam pattern, like Bruker’s smartbeam.^99,103^ Continuous rotation of one diffraction grating with respect to the other leads to quasi-homogenized laser irradiation of a sample.^103^ For sub 5 μm imaging however, using a Gaussian laser beam combined with a aspheric aberration correction might be superior over homogenized beams in terms of spatial resolution and SNR.^100^

Wavelength (ii, *λ*) is also a property of the laser. Lasers with shorter wavelength have improved spatial resolving power and often photon absorption.^104,105^ In practice this potential gain in resolving power is limited by increases in fragmentation, adduct formation, photochemical reactions^106^, costs, and complexity of the necessary instrumentation with decreasing wavelength, especially below 200 nm. Furthermore, almost every MALDI protocol has been optimized for near ultraviolet wavelengths, such as 337, 349 and 355 nm.

Beam diameter (iii, *D*_*b*_) may be expanded to reduce final laser spot size,^96^ whereas, smaller focal lengths (iv, *f*) and angles of incidence (v, *θ*) improve spatial resolving power. This combination of beam diameter, focal length, and angle of incidence leads to a dilemma for instrumentation scientists.

Ideally, both laser and ion collection optics are placed close to sample and at the sample normal angle. However, laser optics placed directly in front of the sample would interfere with the electrical fields of the ion optics and ablated material would deposit on and obscure the surfaces of the laser optics. In practice, the laser optics are placed at an angle suboptimal for spot size and further away from the sample, thereby sacrificing spatial resolving power in favor of ion transmission and ion source reliability. As a result, the focal spot size in standard reflection geometry MALDI imaging at a wavelength of 355 nm is limited to 4–5 μm.^67,96^ The majority of MALDI imaging instruments operate in this “reflection geometry”, in which the sample irradiation and ion collection are both performed above the sample (Figure 4a top).

**Figure 4:**
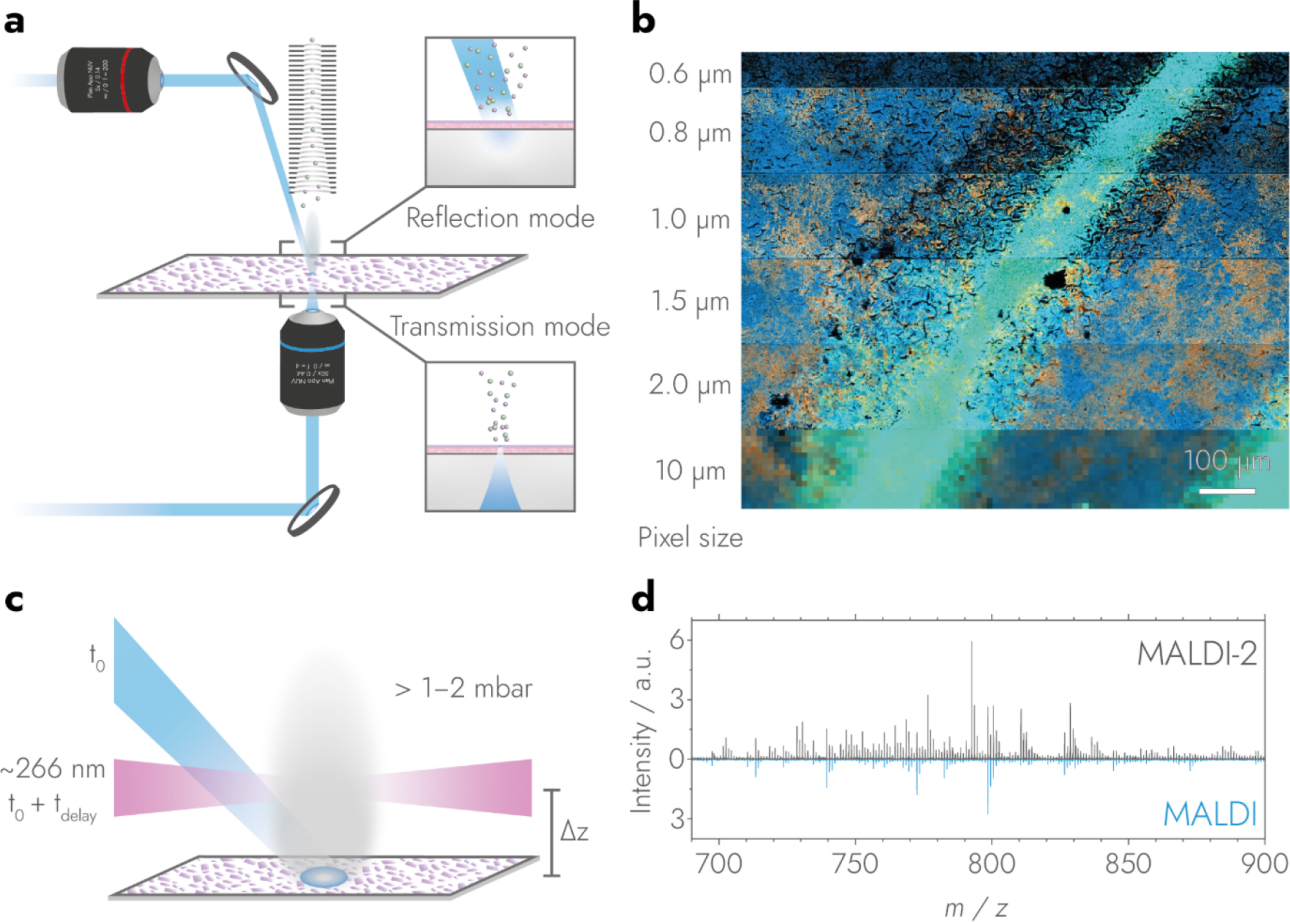
Transmission mode MALDI combined with MALDI-2 enhances spatial resolving power in MALDI MSI. In reflection geometry (a, top) the MALDI laser is focused onto the sample from a larger distance and angle. As a result, laser spot size is usually limited to 4–5 μm using common MALDI wavelengths. In transmission geometry (a, bottom) the laser is focused directly below the microscope slide allowing the use of lens objectives with higher numerical aperture and thus for smaller spot sizes than in reflection geometry. In combination with additional ionization enhancement, for instance via MALDI-2, transmission mode improves spatial resolving power in MALDI MSI (b, adapted with permission from Macmillan Publishers Ltd: NATURE METHODS, Niehaus, M.; Soltwisch, J.; Belov, M. E.; Dreisewerd, K. *Nat. Methods* **2019**, *16*, 925–931 (ref 6). Copyright 2019.). MALDI-2 features a laser ionization step of a MALDI plume at EP (depicted in gray, c) to enhance ion yield by 2–3 orders of magnitude, including ions that were not detectable with MALDI alone (d, adapted with permission from Soltwisch, J.; Kettling, H.; Vens-Cappell, S.; Wiegelmann, M.; Müthing, J.; Dreisewerd, K. Mass Spectrometry Imaging with Laser-Induced Postionization. *Science* (80-. ). **2015**, *348* (6231), 211–215 (ref 112). Copyright 2015 AAAS.).

Alternatively, reflection geometry setups have been built where both ion collection and laser optics are both at the surface normal angle by boring through the center of the laser optics and collecting ions through a dedicated tube.^107^ These setups can achieve spot sizes down to 1.4 μm,^108^ but have never found wide-spread use.

Changing instrumentation geometry from reflection to transmission mode can allow even smaller spot sizes (Figure 4a bottom). Transmission mode was first introduced in 1975 by Hillenkamp et al.,^109^ and later advanced by Zavalin et al. for MALDI imaging.^100^ In transmission mode, a thin sample, such as a tissue section on a transparent glass slide, is irradiated from behind. This mode allows both ion collection and laser focusing optics to be placed at optimum distances and angles without interfering with each other. Laser spots down to 0.6 μm have been achieved using transmission mode MALDI MSI (Figure 4b),^109^ and even higher spatial resolving powers might be feasible as long as the sensitivity of MALDI can be increased sufficiently to compensate for the decrease in sample volume.^6^ The disadvantage of transmission mode MALDI is that the sample is fully ablated preventing subsequent measurements on the same tissue section with other modalities.

#### MALDI-2

MALDI-2 adds a second laser ionization step to MALDI.^110^ Soltwisch et al. discovered that irradiating a MALDI plume with a second UV-C laser, while operating the MALDI ion source at EP, leads to an increase in ion yield by two to three orders of magnitude for many protonated and deprotonated ion species (Figure 4c and d).^89,110,111^ Another approximately twofold ion signal enhancement can be gained by reflecting and refocusing this laser beam onto the MALDI plume for a 2^nd^ time.^112^ Moreover, many molecular species, which were not detectable with MALDI due to ion suppression, ionize readily with MALDI-2 thus expanding the applicability of MALDI.^110^ The discovery of MALDI-2 presents a major breakthrough for MALDI MSI as well as it provides the necessary increase in sensitivity to achieve spatial resolving powers around 1 μm.^6,113^

Laser postionization of UV-MALDI had been attempted several decades ago but led to increased fragmentation.^114^ The main difference to previous experiments is that MALDI-2 operates at elevated pressures,^110^ and, with increased fragmentation, at AP,^94^ instead of high vacuum. The underlying mechanism of MALDI-2 has not been fully explained yet, but three possible mechanisms, 1+1 resonance-enhanced multiphoton ionization (REMPI), REMPI-induced proton transfer (PTR), declustering, or a combination of those have been discussed:

In 1+1 REMPI molecules that absorb at the wavelength of the REMPI laser are first excited into an intermediate state followed by the absorption of a 2^nd^ photon and ionization. REMPI and MALDI-2 both have a wavelength threshold with particularly high MALDI-2 ion signal being obtained above the energy threshold for resonant two-photon ionization of the employed matrix.^110^ Furthermore, the signal enhancement of MALDI-2 benefits from shorter laser pulse widths and rises quadratically with the MALDI-2 laser pulse energy until optical saturation is reached.^89,115^ These findings indicate that MALDI-2 also contains a two-photon absorption step.^115^ However, REMPI ionizes molecules selectively, meaning that at wavelengths commonly used for MALDI-2, like 266 nm, aromatic molecules should be ionized stronger than compounds that do not absorb in this wavelength range.^116^ Furthermore, REMPI leads primarily to radical cations, while MALDI-2 predominantly yields protonated and deprotonated species and only to a marginal extent radical ions and salt adducts.^89,110^ Moreover, the applicability of REMPI to larger biomolecules is limited,^114^ REMPI does not account for variations in MALDI-2 ionization efficiency with matrix choice,^89^ and REMPI is routinely performed in high vacuum, while MALDI-2 requires EP. Thus, REMPI alone is an insufficient explanation for the mechanism of MALDI-2. In fact, Sarretto et al. demonstrated that besides MALDI-2, MALD-REMPI can be performed using low MALDI laser energies and a MALDI-2 wavelength of 266 nm to preferentially ionize aromatic molecules.^117^

A different explanation of MALDI-2 is that REMPI primarily ionizes UV-absorbing molecules, in particular matrix molecules *m*, which subsequently collide with other molecules, causing the formation of protonated matrix and subsequently analyte cations *a*:

2 $$m+2\;h\nu \to m^{+•}$$

3 $$m^{+•}+m\to {\left[m+H\right]}^{+}+{\left[m-H\right]}^{•}$$

4 $${\left[m+H\right]}^{+}+a\to {\left[a+H\right]}^{+}+m$$

For negative polarity, Potthoff et al. suggested that dehydrogenated matrix molecules are ionized via free electron capture (eq 5). We, however, find this capture unlikely considering the high speed and low collision-cross section of free electrons in the gas phase and the absence of charge on the matrix molecule. Instead, we suggest in eq 6 that the electron could also be abstracted from another matrix molecule. Last, the negative charge is transferred from the matrix to analyte molecules, as seen in eq 7.^89^

5 $${\left[m-H\right]}^{•}+e^{-}\to {\left[m-H\right]}^{-}$$

6 $${\left[m-H\right]}^{•}+m\to {\left[m-H\right]}^{-}+m^{+•}$$

7 $${\left[m-H\right]}^{-}+a\to {\left[a-H\right]}^{-}+m$$

Gas phase protonation and deprotonation is a known phenomenon in MALDI without postionization.^39^ The proposed ionization pathway would explain why prevalently protonated and deprotonated species experience an increase in ion intensity in MALDI-2 and why this increase varies with matrix. The mechanism of the reaction shown in eq 2 has a wavelength threshold, and is dependent on laser wavelength, energy and pulse width as described above. Furthermore, the requirement of MALDI-2 for elevated pressures is explained by the REMPI-induced PTR model because higher pressures prolong the lifetime of the MALDI plume as a dense reactive environment, which fosters gas phase reactions.^89,110^

Remarkably, the REMPI-induced PTR model resembles chemical ionization (CI) with the matrix acting as a photo-activated dopant. Bookmeyer et al. found that ion intensity enhancements of protonated species similar to MALDI-2 are reached by single-photon-induced postionization (SPICI) of a MALDI plume using three vacuum UV lamps and acetone as a photoactivatable dopant instead of a MALDI-2 laser.^118^

Although the second model of REMPI-induced PTR is in good agreement with most experimental results, there are findings that allow raising several objections:

First, MALDI-2 was reported to also work for IR-MALDI, albeit with weaker signal enhancement than observed for MALDI-2.^119^ The used matrix, endogenous water, does not absorb UV light strongly, but the investigated samples contained aromatic, UV absorbing molecules, which might have caused enough gas phase (de-)­protonation to cause a signal increase. Moreover, it is unclear whether IR-MALDI-2 and MALDI-2 have the same ionization pathway.

Second, in the REMPI-induced PTR model, molecules with larger proton affinities should presumably yield more ions in both IR and UV-MALDI-2. However, Barré et al. could not find any correlation between MALDI-2 signal intensity and analyte proton affinity.^111^ Still, proton affinity assumes a thermodynamic equilibrium, a condition not fulfilled by the temporally short, energetically activated environment of the MALDI plume.

Third, MALDI-2 works better for higher laser ablation pulse energies than normally used for MALDI, and requires delay times in the order of tens of microseconds between firing both lasers.^89^ The REMPI-induced PTR model alone does not account for these observations.

The third debated mechanism for MALDI-2 suggests that the second laser causes the dissociation and ejection of ions and neutral molecules from clusters formed by the initial MALDI process.^89^ Cluster formation in MALDI is more pronounced at higher laser ablation energies (see ‘spot size effect’) and with IR lasers.^119,120^ Thus, declustering would explain IR-MALDI as well as the requirements for higher laser ablation energies and longer delay times given the slow speed of heavy clusters. However, declustering should not require a wavelength threshold or be a two-photon absorption process. Ejection of ions from clusters also cannot explain the need for elevated pressures or the strong enhancement of (de-)­protonated species in comparison to salt adducts. Thus, the direct ejection of ions from MALDI clusters is an unlikely explanation for MALDI-2. Still, a possibility could be that clusters emit neutral molecules as they cool, and that these molecules are then ionized via REMPI-induced PTR.

### Laser Ablation Inductively Coupled Plasma (LA-ICP) MSI

Laser ablation inductively coupled plasma (LA-ICP) MSI is an ambient elemental imaging method, which was initially developed for the analysis of geological samples and later used to monitor elements in tissue, for instance to localize drugs containing a metal.^121^ Nowadays a rapidly growing application area of LA-ICP MSI is imaging mass cytometry (IMC).^122^

Figure 5 shows a LA-ICP setup where, first, a high-power laser beam irradiates a solid, matrix-free sample causing ablation and the formation of plasma above the sample. Next, the plasma cools, condenses, aerosolizes, and is transported to the ICP ion source by a carrier gas. The ICP coil generates a plasma discharge to atomize and ionize most elements, including most metals with an efficiency >90%.^123,124^

**Figure 5:**
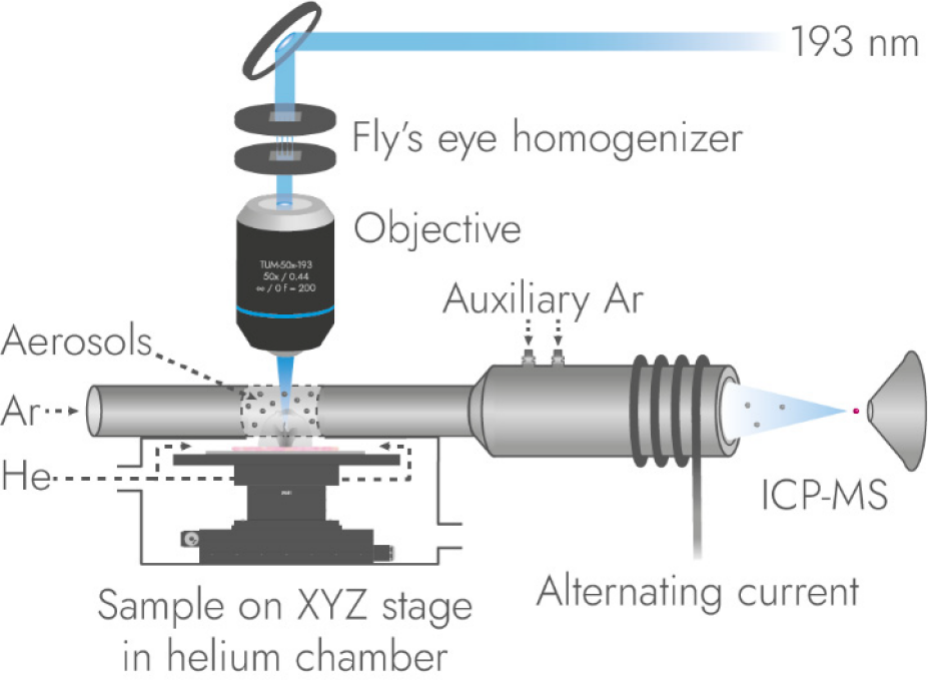
Example of a LA-ICP MSI setup. A high energy 193 nm laser beam is homogenized and focused onto the sample under helium atmosphere, causing ablation. The emitted aerosols disperse in the chamber and are transported by a laminar argon flow to the ICP torch where they are ionized and transferred into a mass spectrometer.

LA-ICP is free of ion suppression effects and has a linear dynamic range of up to nine orders of magnitude.^123^ LA-ICP MSI has achieved a spatial resolving power of 1 μm.^125^ Higher spatial resolutions can be reached by oversampling.^126,127^

The main drawbacks of LA-ICP MSI have been high argon gas consumption and low throughput.^128^ Every laser pulse causes the ablation of aerosol particles of varying temperature, size, composition, volatility, enthalpy of formation, and mass. Particles with different properties behave differently, for instance smaller particles travel faster than heavier ones, which also are more likely to adsorb. Thus, some aerosols are not immediately ionized but stay in the ablation cell’s atmosphere, leading to pixel carryover, sometimes for many seconds, and poorer performance. Thus, a “washout” time of up to several tens of seconds is required after every pixel.^129^ Washout times in LA-ICP-MS can be reduced by adjusting laser beam properties,^130,131^ carrier gas,^132^ ablation cell design,^126–128,133,134^ and aerosol transport to the ICP ion source.^135^ In combination, these adjustments have dramatically increased LA-ICP MSI’s throughput from ≤ 1 Hz to several 100 Hz:

Starting with laser properties, Guillong et al. showed that using a 193 nm wavelength laser combined with an inert gas atmosphere leads to a narrower particle size distribution and overall smaller, easier ionizable, ablated particles compared to larger wavelengths.^130^ Next, laser beam homogenizers are commonly used to achieve uniform laser ablation and particle distributions.^125,136^ Another important parameter is the laser beam pulse width. Nanosecond (ns) lasers can cause cracks in the material, and a “heat affected zone”, in which material melts, redistributes and resolidifyes.^131,137^ Shockwaves and aerosol redeposition lead to debris on the sample around the ablation crater, which can cause carry-over between pixels.^132^ Moreover, “plasma shielding” may occur, in which plasma generated by laser irradiation partly absorbs the later end of the nanosecond laser pulse broadening aerosol particle size distribution and composition.^131,137^ In comparison to nanosecond lasers, femtosecond lasers avoid thermal effects and “plasma shielding”, and allow for higher sensitivity and reproducibility as well as smaller particle sizes that are more representative of the bulk material.^131,137^ Despite these advantages, most LA-ICP MSI systems still operate with ns lasers, most likely because they are cheaper and easier to handle.

Specifically when using ns lasers operating at 193 nm, the carrier gas that transports the aerosol to the ICP ion source influences the signal intensity and the particle size distribution. Air can be used but is not common due a decrease in ion yield and the need for an air to argon gas exchange prior to the ICP ion source.^138^ Argon, which is used to ignite the plasma, would be the natural choice. However, it was shown that using helium instead of argon leads to less debris around the ablation crater, and to more and smaller particles.^139,140^ Horn et al. attributed this effect to the larger heat conductance of helium, which allows heat to be dissipated in the ablation plume more effectively and to thereby quench the formation of larger particles.^140^ This effect is not observable at higher wavelengths or when using femtosecond lasers.^131,140^

The strongest reduction of washout times has been achieved by the design of improved tube cells for laser ablation by the Günther and Vanhaecke groups.^126–128,133,134^ Both labs tried to minimize aerosol dispersion by confining the ablation plume using novel ablation cells with as little volume as possible and that maintain laminar gas flow.^126–128,134^ Furthermore, the sample is not directly exposed to the carrier gas flow. Instead, an auxiliary helium gas flow can be used to orthogonally extract ablated particles and carry them into the path of the carrier gas.^128,134^

Improved cell designs have enabled removing 99% of the residual aerosols of each individual mass peak within less than a millisecond.^134,135^ Still, the total washout time per pixel is >1 ms as heavier atoms and aerosols are slower and arrive after those with lighter mass.^134^

Washout times can also be reduced by optimizing aerosol transport between ablation cell and ion source. Van Acker and co-workers developed an “aerosol rapid introduction system” (ARIS) that avoids pressure losses by using a reduced, constant inner hose diameter leading to increased flow rate and better preservation of laminar flow.^135^ ARIS has enabled LA-ICP MSI at acquisition rates of several 100 Hz.^135^

### Laser Ablation Electrospray Ionization (LAESI) and Infrared Matrix-Assisted Laser Desorption Electrospray Ionization (IR-MALDESI) MSI

Laser ablation electrospray ionization (LAESI) and infrared matrix-assisted laser desorption electrospray ionization (IR-MALDESI) are untargeted, ambient techniques that require little to no sample preparation. Both similarly make use of a high-energy mid-IR laser that can excite the O-H stretching band of water, which if sufficiently abundant, acts as an indigenous IR-MALDI matrix. The ablated analytes are intersected with an electrospray jet to produce multiple-charged ions.^141,142^ The sensitivity of LAESI is generally poorer than that of MALDI.^143^ Sensitivity is enhanced by the application of an IR-absorbing matrix^143^ as well as by using shorter laser pulses.^144^ The spatial resolving power of LAESI MSI can be as good as 40 μm.^145^ Similar to other laser-based approaches, spatial resolving power is constrained by laser focus and ablation crater size, and sensitivity.^141^ LAESI can be used for depth and 3D scans with a depth resolving power of ∼10 μm depending on the amount of ablated and ionized material.^146^ The throughput of LAESI MSI is currently approximately 2 scans s^‑1^.^141^ Analogous to LA-ICP-MS, setups using ablation cells have been explored to reduce washout times and remove geometrical constraints, but these setups have not yet reached the same sensitivity as LAESI setups without ablation cell.^147^ LAESI MSI has the potential to be useful for the in-situ analysis of volatile compounds in water-rich samples, such as plants. We, however, refrain here from an extensive coverage of LAESI MSI as its applicability has been limited and improvements in throughput, sensitivity, spatial resolving power, and reproducibility are needed.^30,148^

## Charged Particle-Based Ionization

### Secondary Ion Mass Spectrometry (SIMS)

#### Introduction and Theory

Secondary ion mass spectrometry (SIMS) is a sensitive, ultra-high vacuum (UHV) MSI technique, which is used to map elements and molecules in tissues and cells. Unlike MALDI, the mass range of SIMS, as a generally hard ionization technique, is usually limited to <2 kDa. However, SIMS offers the highest lateral and depth resolving power of all common MSI methods achieving spatial resolving powers of 0.5-2 μm for molecules and <20 nm for elements,^7,149–151^ as well as depth resolving powers below 10-20 nm.^35,150^

In SIMS, a primary ion beam is accelerated by an electric field of up to tens of keV and focused onto a surface (Figure 6a). The high-energy primary elemental ions penetrate into the sample, fragment molecules that are directly impacted, and transfer momentum to adjacent atoms and molecules, which in turn redistribute the energy of the primary ion further in a collision-cascade (Figure 6b). Atoms, fragments of molecules, and some intact molecules in the first few monolayers of the sample can therefore possess enough internal energy to desorb. The exact ionization mechanism of SIMS has not been explained by a single model. It is likely that a variety of different mechanisms, both on the sample surface as well as in the gas phase, induce ionization. Information on these models can be found elsewhere.^7,152,153^

**Figure 6:**
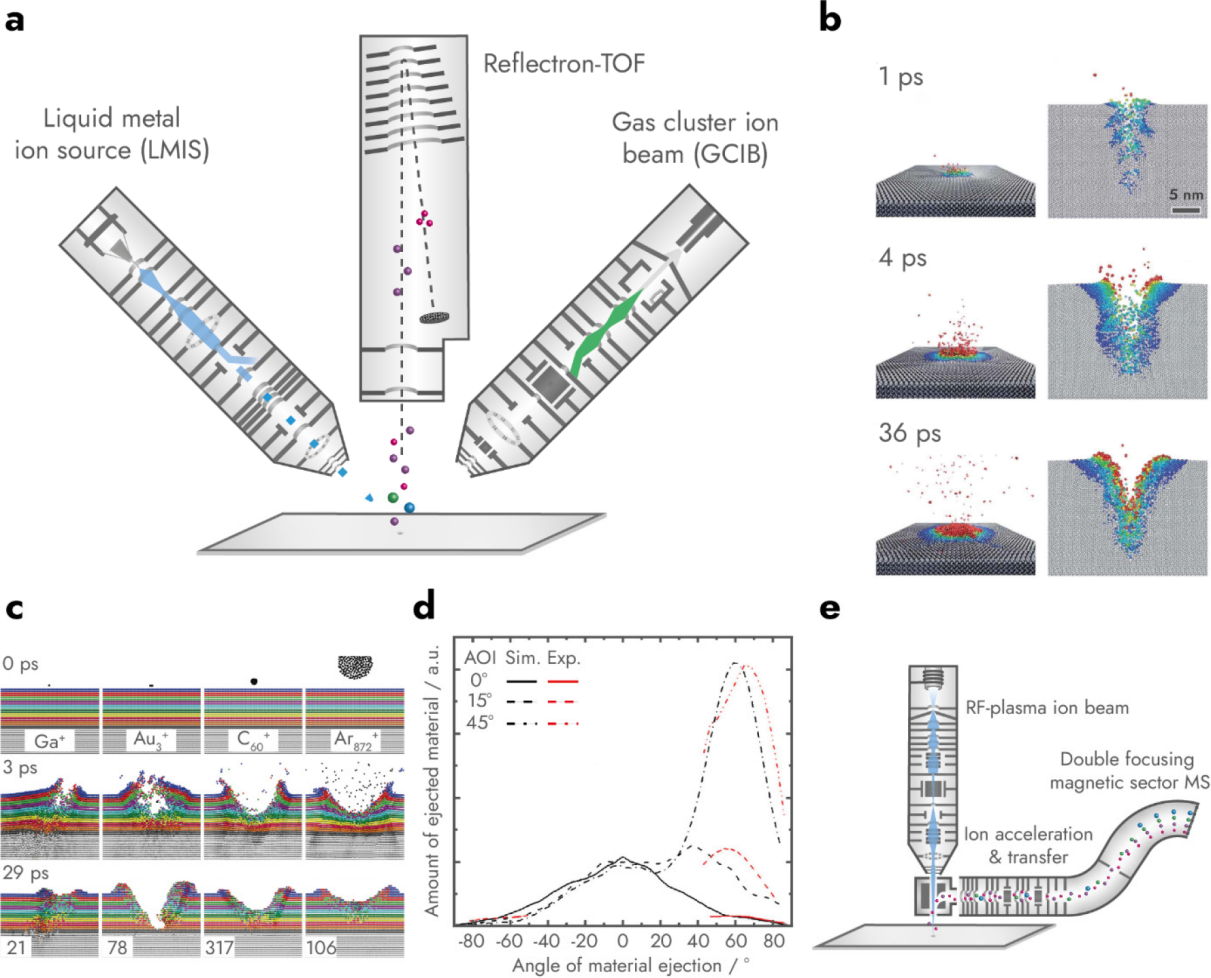
A secondary ion mass spectrometry (SIMS, a) setup drawn with a liquid metal ion source (LMIS) and a reflectron time-of-flight (TOF) mass spectrometer. Primary ion pulses from the LMIS are accelerated and focused onto the surface where they cause the emission of secondary ions. Next to the LMIS, gas cluster ion beams (GCIBs) are commonly used for removing sample layers. As GCIBs cannot be pulsed effectively, using them for SIMS analysis would require a different mass spectrometer, for instance an orthogonal TOF or an Orbitrap. In (b) a molecular dynamics simulation (adapted with permission from Computational View of Surface Based Organic Mass Spectrometry, Garrison, B. J.; Postawa, Z. *Mass Spectrom. Rev*. Vol. 27, Issue 4 (ref 154). Copyright 2008 Wiley.) shows the impact of a primary Au^+^ ion at different time points. The ion perforates the surface and causes the displacement of atoms and molecules in a collision cascade. Material from the upper layers can also be ejected and, when charged, be detected with MS. The colors refer to the degree of displacement: red >2 nm, yellow 1.6 nm, green 1.2 nm, cyan 0.8 nm, blue 0.4 nm, and gray <0.4 nm. In (c) molecular dynamics simulations show Ga^+^, Au_3_^+^, C_60_^+^, and Ar_872_^+^ primary ions hitting a surface at different times after primary ion impact (adapted with permission from Vickerman, J. C.; Briggs, D. *TOF-SIMS: MATERIALS ANALYSIS BY MASS SPECTROMETRY*, 2^nd^ ed.; IM Publications LLP and SurfaceSpectra Limited, 2013 (ref 155). Copyright 2013 Surface Spectra.). The numbers in the lower row indicate the amount of sputtered material for each primary ion. Primary cluster ions cause less subsurface damage and fragmentation while achieving higher sputtering yields than monatomic primary ions. Furthermore, Sputtering yield and direction with gas cluster ion beams depend on the primary ion angle of incidence (AOI, d, adapted with permission from Kański, M.; Postawa, Z. Effect of the Impact Angle on the Kinetic Energy and Angular Distributions of β-Carotene Sputtered by 15 KeV Ar2000 Projectiles. *Anal. Chem*. **2019**, *91* (14), 9161–9167 (ref 156). Copyright 2019 American Chemical Society.). Unlike other ion beams, highest sputter yield for Ar_2000_^+^ is achieved at 45° AOI and ∼60° angle of ion collection. In (e) a nanoscale SIMS is depicted, in which primary ions pass through the same ion optic as secondary ions to achieve nanoscale ion beam focusing by having a short working distance to the sample and an angle of 0° to the surface normal.

The surface damage caused by SIMS can induce alterations in subsequent, spatially-nearby mass spectra, particularly for molecular species due to fragmentation. Thus, for organic analysis it was preferable to either remove damaged layers or to probe every spot on the sample only once, termed “static SIMS”. In static SIMS the total primary ion dose is limited to maximal 10^13^ ions cm^‑2^ leading to <1% of the uppermost surface layers being damaged.^4,149,157^ Using higher primary ion doses, for example by increasing ion gun current or by firing continuously instead of pulsing, termed “dynamic SIMS”, was reserved for the removal of volumes for the analysis of inorganic species. However, the emergence of polyatomic ion guns, like fullerene and gas cluster ion beams (GCIB), with reduced sample damage and improved sputter yields to remove sample debris have improved 3D volume SIMS.^149,153,157,158^ As a consequence, larger volumes of organic surfaces can be sputtered and ionized, which greatly increases sensitivity.^158^

#### Sample Preparation

##### General Considerations

In SIMS, all samples must be UHV compatible (see MALDI section on sample preparation), and, for microprobe-mode, have minimal surface-height variations.^159^ Samples containing volatile molecules, for instance water, have to be freeze-dried or cooled to cryogenic temperatures prior to inserting the sample into the mass spectrometer.^160^ For elemental SIMS MSI of organic tissue, chemical fixation is also possible as long as the sample morphology is preserved.^159,161^ In any case, for biological tissues, samples should be washed to remove salts as those can cause ion suppression.^159,160^

The impact of primary ions leads to the accumulation of charge on surfaces with poor conductivity, e.g. biological tissues. This charge accumulation can distort the electric fields needed for secondary ion extraction. Thus, it is necessary to put insulating samples onto conductive supports, for instance ITO-coated glass slides, or beneath a thin metal mesh. If charge-effects are still present, the sample can be metallized, for instance by coating with gold, or irradiated with a low energy electron gun for positive charge neutralization.

##### Cryo-SIMS

SIMS of cryogenically cooled samples has gained popularity following technological advances in sample preparation and vitrification by the cryo-EM community,^162,163^ and by the introduction of new SIMS instruments with cryogenic capabilities.^164^ Cryo-SIMS allows analyzing semi-volatile species that otherwise would evaporate off under UHV,^165^ leads to reduced fragmentation due to a decrease in internal energy,^165^ and enhances sensitivity in water-rich samples as water enhances ionization via proton transfer reactions.^166,167^

Samples in cryo-SIMS are often prepared by plunging tissue into liquid nitrogen.^168^ This is possible, but leads to ice crystal formation and subsequent tissue damage, which is a particular issue when studying single cells.^162^ Ice crystal formation can be avoided by cooling the sample rapidly at rates of 10^5^-10^8^ K/s and by continuously keeping the sample at temperatures below 136 K.^163^ The most common methods for rapid sample cooling are high-pressure freezing (HFP), in which during cooling with liquid nitrogen the pressure on the sample is gradually increased to ∼2000 bar to lower the melting point of water, and, for < 1 μm thin samples, plunge freezing, in which the sample is rapidly immersed in liquid ethane, propane, or a mixture of both.^162^ The sample is then kept at low, ideally constant, temperature and sometimes treated with a cryo-protectant, e.g. ammonium formate.^166^

##### Matrix Enhanced (ME-)­SIMS

Adding a matrix, for instance one that is commonly used for MALDI, to the sample can increase ion yield of some molecular species, like phosphatidylcholine and sphingomyelin,^169^ by up to 1-2 orders of magnitude and mitigate molecular fragmentation. The degree of signal enhancement varies strongly with matrix, analyte, its chemical environment, and the analyte-to-matrix ratio, for which different optima have been reported.^170–172^

The mechanism of ME-SIMS is not yet fully understood. The mechanism must be different than in MALDI due to the absence of laser light and because a reactive plume is not formed. The main theory for ME-SIMS is that acidic (or basic) matrices enhance the formation of pre-charged analytes on the surface in analogy to “lucky survivors” in MALDI.^170^ In this theory’s support, Wu et al. observed a depletion in cation signal when increasing the pH value to seven.^170^ However, a signal increase is not observed for all species,^77^ and ME-SIMS with acidic matrices also leads to some signal increase in negative polarity.^71^ Thus, other effects could exist, for instance that the matrix serves as protective encapsulation to avoid fragmentation,^171^ that the matrix surface sputters more atoms per ion impact,^173^ that clusters are sputtered and decluster in the gas phase,^173^ or that some organic compounds are selectively enriched in the matrix layer.^77^

##### Metal Assisted (Meta-)­SIMS

Another way that was shown to enhance positive ion yield in SIMS of standards by at least an order of magnitude, is to plasma coat a sample with a metal layer that is usually 1-2 nm thick.^169,174^ MetA-SIMS is useful to image small lipids,^169,175^ can be more reliable than ME-SIMS,^173^ but the obtained spectra are more complicated due to the formation of metal-adducts and clusters. Most often gold is used due to its chemical inertness, good cationizing properties and monoisotopic nature.^176^

Sample metallization makes insulating surfaces conductive and helps prevent local surface charge buildup. However, increased conductivity alone does not account for the observed signal enhancement, which for example is not observed with an aluminum coating.^176^ One theory is that gold surfaces have a higher nuclear stopping power, i.e. atomic density, than organic surfaces.^174^ Higher nuclear stopping power causes more of the primary ion’s momentum to be lost near the surface, which increases sputtering and reduces fragmentation.^174^ But this does not explain why MetA-SIMS does not work in negative polarity mode, or why cholesterol experiences an especial boost in signal.^175^ Moreover, plasma coating does not lead to the formation of a uniform metal layer, which would be too thick for analyte ions to desorb efficiently, but that metal islands form on the surface.^174,176,177^ Delcorte et al. showed that molecules diffuse onto metal islands, with small molecules diffusing faster than large ones.^174^ Molecular diffusion and enrichment accounts for the ion-selective enhancement of MetA-SIMS. It also explains why freshly metallized samples exhibit time-dependent MetA-SIMS enhancements until the metal islands are covered by several monolayers and why enhancement can be partially restored by sputtering away these top layers.^174^ In addition to the above described processes, Delcorte et al. proposed an associative ionization mechanism above the surface, in which an excited metal atom forms a complex with a molecule and stabilizes itself by ejecting an electron.^174,177^ This mechanism can account for the increased observation of metal-adducts and only works in positive polarity. Furthermore, it might explain why MetA-SIMS works for excited d-shell atoms, and not for excited p-shell metals like aluminum, because excited metal atoms with holes in the d-orbitals are better shielded by filled outer s- and p-orbitals.^176^

#### Sensitivity

Sensitivity in molecular SIMS is impaired by ion suppression effects and a decrease in secondary ion yield with increasing molecular weight. In addition to previously discussed topics, such as sample preparation, sensitivity is influenced by a number of instrument parameters, like primary ion beam current, sputter yield, ionization efficiency, and, discussed in mass analysis section, ion transmission and detection efficiency of the mass spectrometer.^7^

First, primary ion beam current depends on type and operating mode of the ion gun. Continuously operating ion guns (“DC mode”) produce higher beam currents than pulsed ion beams, but require different mass analyzer designs. In pulsed-mode, primary ion beam current increases with repetition rate and pulse width of the primary ion beam.

Second, sputter yield increases with primary ion current, mass, size, and up to a point, kinetic energy.^178,179^ Above this maximum kinetic energy point, primary ions penetrate too deeply into the sample and the upper layers are less affected by the collision cascade.^7,179^ A benefit of higher kinetic energies is an increase in intensity of molecular high-mass ion signals.^178^ Cluster ion beams generally achieve higher sputtering yields and cause less fragmentation than monatomic ion beams (Figure 6 **c**).^153,158^ This is because clusters dissociate upon impacting the surface into multiple atoms or small molecules with each carrying only a small fraction of the cluster’s momentum. Sputter yield is generally higher at large angles of primary ion incidence to the sample normal,^7^ while the angle at which most secondary ions are emitted, and at which ion collection should ideally be done, depends on nature, kinetic energy and angle of incidence of the primary ion beam.^180^ For example, monatomic argon and fullerene ions have been shown to eject most material at angles between 0 and 20° with little dependence on the angle of incidence, whereas the sputter yield using large cluster projectiles is maximal at an angle of incidence of 45° with most matter being ejected at angles around 60° (Figure 6d).^156,180,181^

Third, SIMS ionization probability per sputtered particle is typically only in the order of 10^‑3^-10^‑5^.^182^ A strategy to enhance ionization efficiency is the implementation of an additional laser ionization step. For elemental SIMS this results in a two orders of magnitude improvement in ion yield and mass spectra more representative of the sample.^183^ For molecular SIMS however, an extra laser ionization step induces additional fragmentation, and to date no method similar to MALDI-2 exists yet for SIMS. Gilmore et al. summarized progress in this research area in another review.^149^ Higher ionization efficiencies can also be achieved by using reactive primary ion species. For example, cesium and oxygen produce elemental anions and cations, respectively, and water clusters enhance molecular SIMS through de-/protonation.^7,153^ Alternatively, cesium and oxygen can be flooded into the chamber, and water clusters can be deposited on the sample prior to analysis.^7,167,184^

#### General Considerations on Lateral and Depth Resolving Power

Spatial resolving power in microprobe-mode SIMS is determined by sensitivity, spot size of the primary ion beam, and by the amount of lateral spread of the collision cascade, which decreases for heavier primary ions.^7,185,186^ General methods to improve primary ion beam focus are to (i) reduce Coulomb interactions and the energy spread they cause, by decreasing beam current at the expense of sensitivity or analysis time, (ii) mitigate spherical aberrations of the focusing lens by blocking the outer parts of the primary ion beam with an aperture, (iii) irradiate the sample at a 0° angle to the surface normal to avoid obtaining an elongated, elliptical spot, (iv) keep the working distance of the focusing ion optics as short as possible.^7,159,187^ Highest lateral resolving power elemental SIMS instruments, like Cameca’s NanoSIMS, therefore focus a DC primary ion beam through the same ion optical assembly used for extracting secondary ions.^150,159,185^ This setup in conjunction with a double focusing sector mass spectrometer allows for elemental SIMS with lateral resolving powers of 50,^159^ 37,^188^ <20,^150^ and ≤15 nm,^185^ using Cs^+^, O^‑^ radiofrequency plasma, Ga^+^, and He^+^ gas field ionization ion beams, respectively (Figure 6e).

For molecular SIMS the same considerations are valid but different ion guns and mass analyzers are used. In practice, spatial resolving power is then limited by sensitivity and therefore by the brightness of the used ion gun.

Depth resolving power in SIMS is mainly sample and ion gun-dependent. Monatomic primary ions at low kinetic energies achieve resolutions below 10 nm,^150^ but cause substantial damage to organic samples. Rotating the sample and choosing a grazing incidence angle can mitigate these effects. Nowadays depth and 3D scans of organics are usually done with GCIBs, which cause less surface roughening and sputter more efficiently than monatomic ion guns.^35,158^ GCIBs enable sputter rate depth resolutions down to <20 nm.^35^ As the lateral focus size of GCIBs is at best limited to 3-10 μm,^189–191^ a common practice is to use a GCIB for sputtering and removing subsurface damage and another ion gun for high-resolution imaging.^192^

#### Ion Guns

Ion gun selection is an important part of an optimal SIMS experiment. Table 1 provides some comparison metrics, where available, for ion guns commonly used in SIMS. The most important parameter for building a well-focused ion beam to smaller spots at higher currents is the reduced brightness *B*_*r*_, which we define here for a point source as:^187,193^

8 $$B_{r}=\frac{I}{A\Omega V}$$

where *I* is the primary ion current, *A* is the virtual source size of the ion emitter, which is the smallest volume that all particles could have emitted from, given their trajectories, Ω is the solid angle at which particles are emitted, and *V* is the acceleration potential of the ion gun.^187,193^ Reduced brightness is a useful metric as it is a conserved property throughout the column of the ion gun.^187^ The next parameter is primary ion energy spread, which causes chromatic aberrations and limits how finely an ion beam can be focused.^194^ Aberration-correcting ion optics can compensate for higher energy spread, but have not found wide-spread use due to increased complexity and price.^195,196^ Third, increasing the ion gun’s acceleration potential allows achieving higher sputter yields and better primary ion beam focusing.^194^ In practice, the maximum acceleration voltage of an ion gun is limited by cost and stability of power supplies or the possibility of electrical discharge in the ion gun. Besides these figures of merit other parameters, like the operating modes of the ion gun (DC and/or pulsed), minimum achievable pulse duration, source lifetime, primary ion current stability, induced surface damages, and, for molecular SIMS, achievable mass range, are important as well.

**Table 1:** Most Commonly Used Primary Ion Guns Used for SIMS and Their Figures of Merit Found in Literature

	**Typical beam current**	**Virtual source size**	**Reduced brightness**	**Energy spread**	**Typical acceleration potential**
**Cs ion guns**					
Surface ionization^197–199^	Up to 200 nA	50 μm	10^2^ A cm^–2^ sr^–1^ V–	0.2–0.5 eV	10–20 keV
Low temperature ion source (LoTIS)^7,200,201^	1 pA–10 nA	10 μm	10^7^ A cm^–2^ sr^–1^ V^–1^	0.34–0.45 eV	10–30 keV
**Plasma ion guns**					
Duoplasmatron^186,202^	Up to 500 nA	200–300 μm	10^2^ A cm^–2^ sr^–1^ V^–1^	5–20 eV	30 keV
Inductively coupled plasma^186,188,200^					
Xe^+^	0.1 pA–10 μA	35–50 μm	10^4^ A cm^–2^ sr^–1^ V^–1^	5 eV	30 keV
O_2_^+^	0.1 pA–4 μA	35–50 μm	4 × 10^3^ A cm^–2^ sr^–1^ V^–1^	5 eV	30 keV
O^–^	0.1 pA–400 nA	35–50 μm	4 × 10^2^ A cm^–2^ sr^–1^ V^–1^	3.5 eV	30 keV
**Gas field ionization source (GFIS)** ^203^	1 fA–100 pA	3 Å	10^9^ A cm^–2^ sr^–1^ V^–1^	<1	5–45 keV
**Liquid metal ion source (LMIS)** ^186,187,204^					
Ga^+^	0.2 pA–50 nA	3 nm	10^6^ A cm^–2^ sr^–1^ V^–1^	5–10 eV	30 keV
Bi^+^ and Bi_3_^+^ ^193^	2 pA–60 nA	–	–	15–30 eV	30 keV
**Electron ionization based ion guns** ^203^					
C_60_	Up to 2 nA	1 mm	1 A cm^–2^ sr^–1^ V^–1^	–	20–40 keV
Gas cluster ion beams^153,191^	≥5 pA	1 mm	1 A cm^–2^ sr^–1^ V^–1^	–	20–70 keV

##### Cesium Ion Sources

Most cesium ion beams are generated by passing a cesium gas through hot, porous tungsten frit where the cesium atoms undergo surface ionization with approximately 99% ionization efficiency.^198^ This process enables high beam currents with low energy spread. Furthermore, cesium is highly reactive and therefore enhances negative ion formation, making cesium ion beams a good choice for imaging nonmetals, negatively charged polyatomic metal compounds, and low mass organic fragments at spatial resolving powers down to 50 nm.^159^ However, the reduced brightness of the cesium ion gun is poor, which prevents sub 50 nm imaging.^203^

A different approach is the low temperature ion source (LoTIS), in which a neutral cesium beam is laser-cooled to as little as 10 μK prior to resonance-enhanced two-photon ionization.^201^ The resulting ion beam has similar energy spread and is 4-5 orders of magnitude brighter than what is achievable with surface ionization. Steele et al. showed that LoTIS beams can be focused down to approximately 2 nm, which even though no SIMS data using a LoTIS have been published yet, make this ion beam very promising for sub 50 nm SIMS MSI.^201^

##### Plasma Ion Sources

Hot plasmas are used to generate high current ion beams of gases, such as xenon, argon, or oxygen. Oxygen plasma ion guns are especially complementary to cesium ion guns, as they chemically enhance cation formation and are routinely used for high-resolution imaging of metals.^159^ In a Duoplasmatron hot plasma ion gun, a gas is leaked into the gun where it is ionized between three electrodes by arc discharge.^202^ The main disadvantage of Duoplasmatron ion guns is a poor reduced brightness, which makes sub 100-200 nm SIMS imaging with Duoplasmatrons infeasible.^200^ Additionally, the electrodes inside the source deteriorate over time, especially when oxygen is used.^200^ This deterioration limits the ion source lifetime to 50-500 hours and contributes to primary ion current instability.^188,205^

A newer alternative to Duoplasmatrons is to use a radio frequency (RF) inductively coupled plasma (ICP) for ionization.^200,205^ ICP ion guns do not contain material-wearing electrodes in the source and thus have much higher lifetimes (> 2 years).^200^ Furthermore, energy spread of ICP sources is lower while reduced brightness is 1-2 orders of magnitude higher than that of Duoplasmatrons. Consequently, SIMS with ICP ion guns allows for an order of magnitude improvement in sensitivity and higher lateral resolving powers than with Duoplasmatrons. For example, Malherbe et al. demonstrated a spatial resolving power of 37 nm using O^‑^ primary ions.^188^

##### Liquid Metal Ion Source (LMIS)

In a liquid metal ion source (LMIS), also known as liquid metal ion gun (LMIG), a heated, porous, and blunt tungsten needle is wetted with a molten metal and charged. At the tip of the needle a high electric field acts on the liquid metal, causing it to form a Taylor cone with a tip only a few nanometers wide.^204^ The tip of the cone ejects ionized atoms and clusters, of which one species is selected with a mass filter to be the primary ion. The resulting ion source has high reduced brightness due to a small virtual source size.

The focusing capability of LMIS depends on the element being used. Virtual source size and energy spread increase with the mass of the primary ion leading to larger spot sizes, but heavier ions achieve higher sputter yields and experience a higher nuclear stopping potential than lighter species.^179,193^ Gallium is a popular choice, both in SIMS and in focused ion beam scanning electron microscopy (FIB-SEM), due to its high lifetime, low melting point and vapor pressure.^187,193^ Furthermore, a gallium LMIS produces almost exclusively single charged ions and does not require a mass filter in the column of the ion gun, which reduces cost and size of the ion gun.^193^ Ga^+^ guns achieve spot sizes below 10 nm, which is smaller than the collision cascade they induce.^193,203^ Elemental SIMS has been demonstrated with Ga^+^ at resolutions down to 15 nm.^150^ However, the main drawbacks of using Ga^+^ ion beams for SIMS is their low current when focused as well as their low ionization efficiency leading to overall poor sensitivity.^193,200^

Using other metals, for example indium, gold, or bismuth, is therefore sometimes more practical despite worse later resolving power and increased gun complexity. Bismuth and gold produce relatively high amounts of clusters.^193^ These cluster ions exhibit larger energy spread and less current than their monatomic counterparts.^193^ However, they dissociate and split their momentum upon impacting the surface leading to higher sputtering efficiency, reduced surface damage, and allow detecting molecular species up to 800-1000 Da.^4^ The ionization efficiency of these LMIS clusters for such molecular species however, is often only sufficient for an averaged mass spectrum over the entire spatial area rather than imaging.^4,206^

##### Gas Field Ionization Source (GFIS)

In a gas field ionization source (GFIS), helium or neon gas is directed through a cryogenically cooled needle with 100 nm apex radius. This needle is positively biased causing field ionization and therefore the inner shell electrons of the gas to tunnel to the needle, leaving behind positively charged ions.^7^ The emission pattern of a GFIS consists of three atomic beamlets, making GFIS have the smallest virtual source size and the largest reduced brightness of all commonly used ion beams.^203^ Furthermore, primary ions have a small energy spread leading only to negligible chromatic aberrations when focusing.^200^ As a result spot sizes as small as 0.35 nm for helium and 1.9 nm for neon can be achieved.^200^ The spatial resolving power of GFIS SIMS is thus only limited by sensitivity and the size of the collision cascade (∼10 nm). Dowsett et al. demonstrated that sub 20 nm resolving power can be reached using helium^+^ GFIS SIMS.^185^ Next to the need for cryogenic cooling, and pure gas, the disadvantages of GFIS are lower sputtering and secondary ion yield in comparison to gallium^+^ LMIS.^203^ Helium sputters especially poorly at about an order of magnitude less than neon, likely due to helium’s lower nuclear stopping power and unreactive nature.^200,203,207^ Unfortunately other gases are not compatible with the GFIS as they might condense inside the needle and react with the needle material.^200^

##### C_60_ Ion Source

A fullerene (C_60_) ion beam is obtained by sublimating fullerene powder under vacuum followed by electron ionization (EI).^208^ Similar to cluster ions generated with LMIS, C_60_ ions dissociate upon impacting the surface leading to sixty carbon impacts, each with one-sixtieth of the kinetic energy of the fullerene. Consequently, (sub-)­surface damage and secondary ion molecular fragmentation are strongly reduced in comparison to previously discussed ion beams.^157,208^ Thus, C_60_ ion beams allow for higher sputter yields, greater molecular sensitivity and higher observable masses than LMIS.^157^ Due to the relatively low surface damage, C_60_ ion beams allow for effective measurements with ion doses beyond the static limit.^157,158^ In dynamic C_60_ SIMS, a surface damage accumulation is mitigated as most induced changes to the sample, except slow surface carbonization, are sputtered away.^209^

The main downsides of C_60_ ion beams are low current and poor reduced brightness,^203^ which makes it difficult to focus the beam to less than 1 μm while maintaining enough secondary ion yield to record molecular images.

##### Gas Cluster Ion Beams (GCIB)

Gas clusters are formed when gas under high backing pressure is expanded adiabatically into vacuum. The gas cools to cryogenic temperatures as it accelerates towards the vacuum, converting most of its internal to kinetic energy. Most of the gas does not form clusters and is pumped away. However, upon three-body collisions,^181,210^ cluster dimers can form and grow either by monomer addition or cluster aggregationhe resu. The result is a neutral beam with a broad distribution of cluster sizes, with some that have >10,000 monomers.^153,181^ After passing a skimmer, the cluster beam undergoes electron ionization. A Wien filter selects a window of cluster ions, which are then extracted and accelerated.

Upon impacting a surface, gas clusters dissociate into monomers and split their momentum. By tuning cluster size and kinetic energy, the impact energy of a monomer can be less than 1 eV.^211^ For comparison a C_60_^+^ ion at 40 keV dissociates into atoms with a kinetic energy of 667 eV. Thus, gas cluster ion beams (GCIBs) induce less sample damage and molecular fragmentation than all other ion beams making them the most suited ion beam for the analysis of most molecules.^153,212,213^

Consequently, volumes can be sputtered and analyzed with dynamic GCIB-SIMS while static LMIS-SIMS is limited to the upper layers of a sample. In contrast to SIMS with other ion beams, gas clusters may cause surface phenomena, such as interactions between collision cascades,^214^ clusters remaining partially intact on the surface,^211,215^ or matter ejection without prior collision cascade.^181^ The most notable difference to other ion beams, with low secondary ion ejection angles to the sample normal, is that secondary ions in GCIB-SIMS are preferentially ejected at large, forward-directed angles (see Figure 6d).^180,181^

Despite their advantages, gas clusters have many degrees of freedom, high variance in primary ion mass, and are metastable. Furthermore, Wien filters can only isolate large windows of clusters that can vary in size by several 100 monomers.^213^ Thus, it is difficult to pulse GCIBs and nowadays most GCIB SIMS is exclusively performed in DC mode.^216^ High energy spread paired with low brightness limit the ability to focus GCIBs while maintaining enough ion current. Increasing the acceleration potential of GCIBs allows for better ion beam focusing.^189,213^

The gas chosen for cluster production influences the performance of a GCIB. Most commonly used gases are Ar, CO_2_, and, when seeded into a carrier gas, H_2_O.^153^ Clusters consisting of other gases, like O_2_ and N_2_, exist but have not been applied to SIMS yet.^181^ Gas mixtures are employed as well.^153^ Ar clusters were initially most common but have low ionization efficiency and are difficult to focus to less than 10 μm.^189,190^ CO_2_ and H_2_O clusters exhibit less metastable decay in the ion gun column than Ar clusters, and can be focused to about 3-7 μm using acceleration potentials of 40–70 kV.^190,191^

H_2_O cluster beams achieve similar sputter yields as CO_2_ beams at otherwise identical experimental conditions.^215^ However, SIMS with H_2_O GCIBs at <0.15 eV per monomer molecule has higher sensitivity and mass range than CO_2_ and Ar GCIBs, being able to even image molecules in the 1000-1500 Da range.^153,168,211,217^ The ionization improvement of H_2_O cluster ion beams is attributed to the formation of an activated aqueous surface environment, where H_2_O chemically enhance the formation of [M+H]^+^ and [M-H]^‑^ ions.^215,218^

### Desorption Electrospray Ionization (DESI)

Desorption electrospray ionization (DESI) is a soft ambient ionization technique for the analysis of small molecules, lipids, and in some cases proteins and peptides,^219^ on insulating surfaces.^220,221^ In DESI charged electrospray droplets of solvent are supersonically accelerated with a high-pressure gas, e.g. nitrogen, onto a sample causing the formation of a local, wet, analyte-extracting film on the surface.^221^ Subsequent droplet impacts lead to the emission of charged secondary droplets containing analytes from the sample.^222^ These droplets gradually evaporate and ions are collected and transferred to the atmospheric inlet of a mass spectrometer (Figure 7). The resulting mass spectra exhibit both single and multiple charged ions similar to ESI spectra of liquids.^220,222,223^

**Figure 7:**
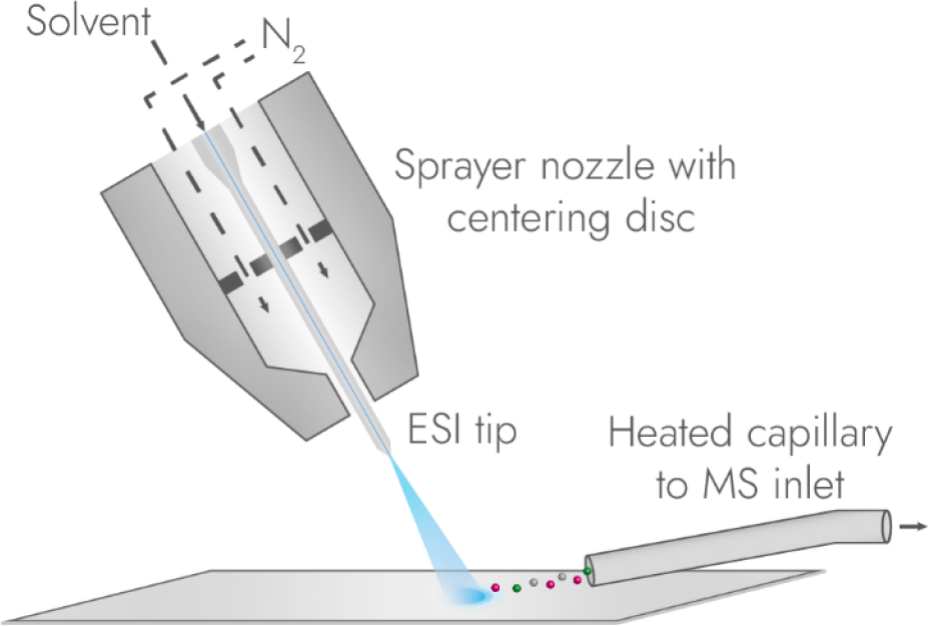
Setup of desorption electrospray ionization (DESI) MSI. The sample is scanned with an ESI tip, which shoots charged droplets onto the surface. There, a wet film forms, which extracts analytes from the sample. Subsequent droplet impacts cause the emission of charged secondary droplets and, following their decomposition, ions. These droplets are collected and transferred to a MS via a heated capillary. Further technical details on the setup of DESI may be found in Table 2.

DESI imaging has several advantages. As it is ambient, samples do not need to be vacuum-compatible, and volatile molecules can readily be analyzed. No sample preparation other than tissue mounting is required. Moreover, some molecules that do not ionize well with MALDI do so with DESI and vice versa.^224^ Although no sample preparation is required, OTCD can be performed either as described in the MALDI chapter or by spraying an OTCD reagent with the solvent in an approach termed “reactive DESI”.^222,225^ Last, DESI MSI is the simplest of all MSI techniques discussed in this review, needing only a sprayer, a movable, automated stage, and a mass spectrometer with atmospheric inlet.

The disadvantages of DESI are low, sensitivity-limited imaging throughput of usually ≤1–5 pixel s^‑1^,^226,227^ low repeatability,^228^ and low spatial resolving power of as good as 50 μm on tissues.^227,229^ Higher lateral resolving power is achievable on heated surfaces and/or on surfaces with low affinity to solvent and analytes, e.g. paper, to facilitate desorption and to prevent solvent spreading.^221,229^ Furthermore, the ideal source geometry is analyte- and surface-height dependent.^221^

Thus, we summarized the settings most commonly used for DESI MSI in Table 2. Some values are trade-offs between spot size and ion yield, for instance solvent flow rate, or the distance between sprayer and sample, where longer distances produce smaller droplets and aid in the ionization of some molecules at the expense of spatial resolving power.^221^

**Table 2:** Common DESI MSI Setting Ranges Found in the Literature

**Setting**		**Value range**
Distances		
	Sprayer – sample	1–5 mm^221,229–231^
	Sample – capillary to MS inlet	0–4 mm^221,230^
Angles		
	Sprayer – sample	50°–80° ^219,221,227,230,231^
	Sample – capillary to MS inlet	5°–10°^221^
Temperature of capillary to MS inlet		450–500 °C^219,227^
Diameter of capillary to MS inlet		500 μm^229,232^
Solvent		–MeOH/H_2_O (50:50–98:2) for lipids^219,230^
		–ACN/H_2_O (80:20) for proteins^219^
		Acidic/basic additives are often added to boost ionization efficiency and adduct formation.^221^
Solvent flow rate		0.5–5 μL/min^221,226,227,229,230,233^
Voltage		4–5 kV^221,229^
Gas pressure		4.5–7 bar^227^

Progress in DESI instrumentation has been achieved by optimizing the design of the sprayer. Initially, conventional, partially self-pulled silicon capillaries of 50 μm diameter were used for spraying,^229,230^ but suffered from poor repeatability due to bending and random motion of the inner capillary with respect to outer gas capillary.^227,228^ The achievable spatial resolving power was only ∼500 μm on tissue, while 40-200 μm have been reported for DESI from paper surfaces.^228–230^ Contrary to an initial report,^221^ reducing the capillary diameter to 10 μm does not substantially improve spatial resolving power and only causes a decrease in ion yield.^232^ Tillner et al. achieved better repeatability and ∼50 μm spatial resolving power using a stiffer 20 μm inner diameter capillary and a central metal disk that fixes the position of the inner capillary (see Figure 7).^227^ Sprayer robustness and to some degree signal intensity can additionally be improved by desorption electro-flow focusing ionization (DEFFI), in which the inner capillary is retracted with respect to the outer gas capillary, and both solvent and gas pass through a small nozzle.^231,234^ This not only temporarily focuses spray droplets to a jet and achieves smaller droplet size distributions, but isolates the sample from the electric field of the sprayer and allows using lower gas pressures and electric fields.^231,234^ The spatial resolving power of this sprayer is also ∼50 μm, but further enhancements seem possible by improved gas focusing and by the optimization of the settings in Table 2, for instance by reducing sprayer to sample distance and solvent flow rate, at the expanse of ion yield.^231^

## Instrumentation

### Mass Analysis

In this chapter the mass analyzers most commonly used in MSI will be discussed from an applied perspective. For more information on the working principles of mass analyzers, we recommend the work by Jürgen Gross.^235^

Important figures of merit of an imaging mass spectrometer are spectra acquisition rate, ion transmission, mass resolving power, accuracy, and mass range. Mass images can consist of millions to billions of pixels, thus the acquisition time of mass spectrum should be as short as possible to avoid impractically low throughput. Ion transmission is crucial as it can limit throughput, pixel size, ability to perform tandem mass spectrometry experiments, and the contrast or quality of individual mass images. High mass resolution, henceforth defined as mass divided by full-width-at-half maximum (M/ΔM_50%_), is particularly important for MSI of complex samples, due to the occurrence of isobaric species.

#### Time-of-Flight Mass Analyzers

The vast majority of MSI is done with time-of-flight (TOF) mass spectrometers. TOFs offer many advantages, such as being able of acquiring entire mass spectra at, depending on chosen mass range and TOF length, rates up to tens of kHz,^236,237^ and are easy to couple to pulsed ionization sources. The mass resolving power of TOFs has improved over the last decades by reducing ion energy spread, increasing ion acceleration voltage, and extending flight path length, which most state-of-the-art TOFs achieve by making use of reflectrons.^67,236,238^ Initial ion kinetic energy spread can be decreased via delayed extraction in axial TOFs,^67^ or by ion beam focusing prior to acceleration orthogonal to its initial flight direction.^236,239^ TOFs with orthogonal acceleration geometry are often termed “oaTOFs” or “QTOFs” in case ion focusing is performed with a quadrupole.^235^ A drawback of oaTOFs is that a fast high voltage pulser is needed to accelerate ions. However, this also enables the coupling of oaTOFs with continuous ion sources.

TOFs offer a combination of high speed and mass resolution, which makes them a good choice for molecular MSI. State-of-the-art, commercially available, axial TOFs reach based on our experience a mass resolution of ≈20,000 between 800 and 900 *m/z*.^67^ Meanwhile MSI with oaTOFs achieves mass resolutions of up to 50,000 in a similar mass range.^240^

Even higher mass resolving powers are achievable by multireflectron (MR-)­TOFs.^241^ In a MR-TOF several electrostatic mirrors or sectors prolong the ions’ flight path to an open zigzag or corkscrew trajectory (Figure 8a).^242,243^ For example, an early MR-TOF already achieved a mass resolving power of 350,000.^243^ However, adaption of MR-TOF technology required overcoming technical limitations: first, ion losses increased with every reflection due to ion packet divergence, aberrations, and, when gridded ion mirrors were used, due to scattering.^238,241,243^ Second, the maximum achievable mass resolving power is limited by ion-optical aberrations, space charge effects, and ion trajectory drift.^241,244^ Third, increased flight paths lead to longer flight times and thereby to reduced duty cycle.^241^ Consequentially, the first MR-TOF MSI instrument achieved only a mass resolving power of ≈30,000 after three reflections while suffering from ion losses.^238^

**Figure 8:**
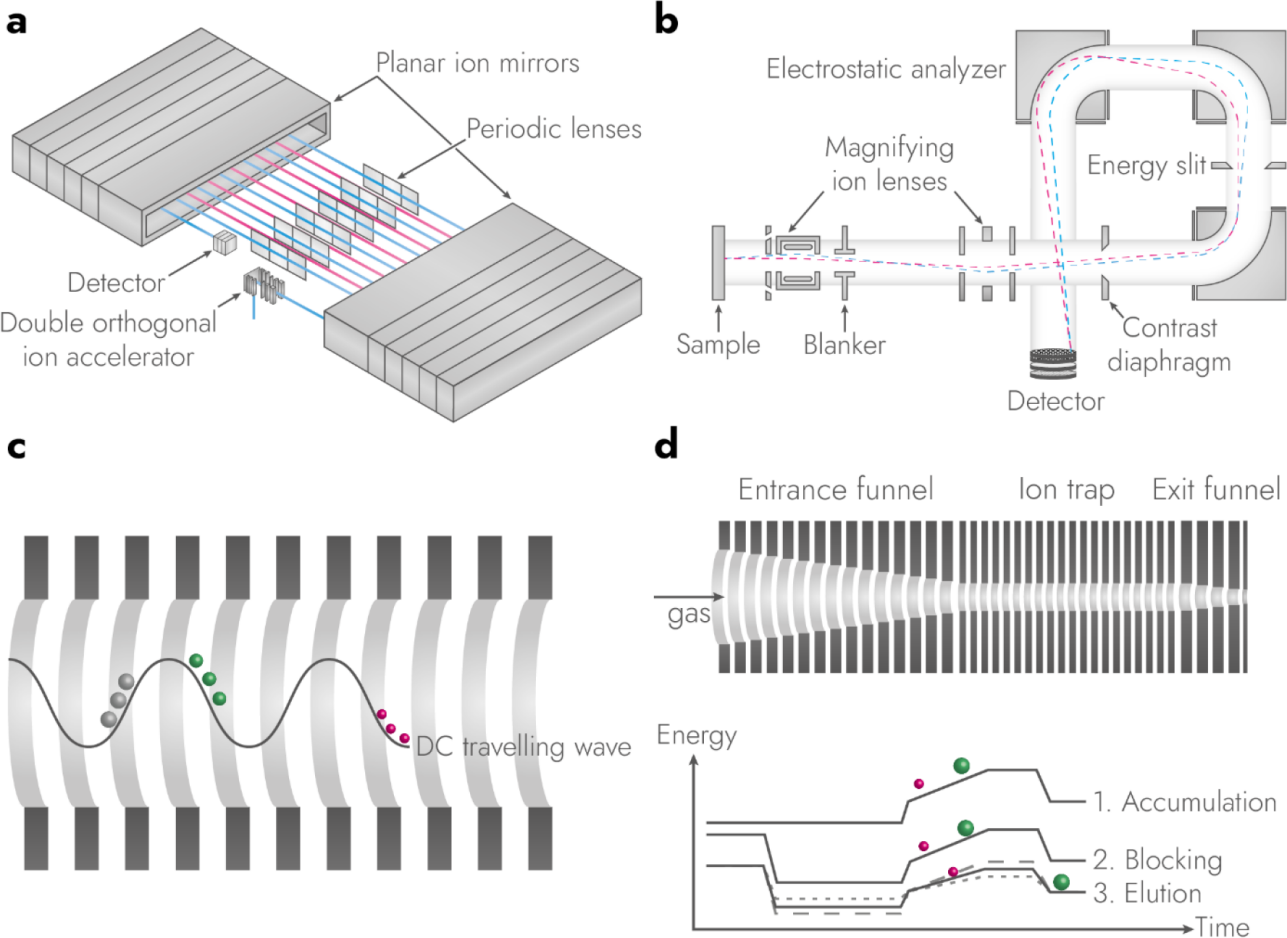
Advanced time-of-flight (TOF) and ion mobility spectrometers. In (a), an open-path multireflectron TOF with planar gridless ion mirrors and refocusing lenses is shown.^249^ In (b), a stigmatic triple ion focusing TOF (TRIFT) is depicted. Analogue to an optical telescope, two lenses magnify an ion image and project it after TOF separation onto a fast pixelated detector. Similar to a reflectron, three electrostatic analyzers equipped with Herzog shunts and Matsuda plates (not shown) elongate the flight path to 2 m and provide 1^st^ order energy correction. Mass and spatial resolving power are limited by aberrations and can be increased at the cost of ion transmission with an energy slit and a contrast diaphragm, respectively.^20,252^ In (c and d), a travelling wave and a trapped ion mobility spectrometer are displayed (TWIMS and TIMS, respectively).^253^ In TWIMS, ions are radially confined and pushed forward by periodic DC waves against a stationary gas. In TIMS, ions are accumulated in an ion trap while a counter directed gas flow prevents ions from propelling ions forward and distributes ions spatially separated in the ion trap. After an accumulation period, the ion trap is blocked against further ions, and the trapping potential is gradually lowered, allowing ions with ever smaller collision-cross section to elude the ion trap. Another TIMS design consists of two ion traps in series, allowing for parallel accumulation in the first and IMS separation in the second ion trap. This design allows for a 100% duty cycle without the need for blocking the TIMS cell, and acquiring multiple MS/MS spectra in one go via PASEF.^253,254^

The performance of MR-TOFs increased substantially after a series of improvements: First, refocusing lenses between gridless mirrors reduced ion losses at the expense of greater vulnerability to space charge effects.^241,244–246^ Alternatively, Grinfeld et al. presented a mass spectrometer that allows for a controlled amount of ion drift between two slightly tilted ion mirrors prior to refocusing ions onto the detector.^244^ Second, the development of planar gridless ion mirror pairs with additional accelerating fields at their entrances provide aberration correction superior to those in previous MR-TOFs.^247,248^ Third, the issue of reduced duty cycle was overcome by allowing ions of different TOF pulses to overlap leading to spectral congestion. In the case of TOF overlap between pulses, encoded frequent pulsing (EFP), in which the time between TOF pulses is varied systematically, is applied followed by deconvolution.^241^

MR-TOF with these improvements were commercialized first for GC-MS and later also for MSI.^249^ MR-TOFs now reach ≈200,000 mass resolving power and sub-ppm accuracy with >10% duty cycle after a 48 m flight path.^249^ Theoretically, higher resolving powers can be obtained by restricting mass range, sending ions through the MR-TOF multiple times in a closed loop, or by extending the flight path while reducing ion package size to minimize space-charge effects.^250^ More information on MR-TOFs as well as an outlook on further advancements can be found in reviews by Verenchikov et al.^241,251^

Some TOFs also have a stigmatic ion imaging capability, which means they can extract, magnify, preserve, and project ion images throughout the mass spectrometer onto a spatially-resolved detector.^16^ However, only the triple ion focusing TOFs I and II (TRIFT I and II, Figure 8b) have ever been commercialized. A TRIFT consists of three electrostatic analyzers (ESAs) which prolong the flight path to 2 m, and, in combination with a contrast diaphragm and an energy slit, reduce ion energy spread leading to higher mass resolving power and smaller chromatic image aberrations.^20,21^ The TRIFT II can achieve mass resolving power of 15,000 at *m/z* > 200, and spatial resolving powers of 2.5 and 3.4 μm for SIMS and MALDI, respectively.^8,23^

Non-commercial advancements include that Aoki et al. achieved an increase in mass resolution similar to delayed ion extraction using post-extraction differential extraction (PEDA). In PEDA, ions are focused in time by increasing the acceleration potential of the TOF after a short time delay after extraction.^255^ This concept was incorporated into a stigmatic reflectron TOF to reach a mass resolving power of ≈8,000.^256^ The spatial resolving power was as good as 20 μm but varied strongly for different m/z values. Attempts to build stigmatic MR-TOFs, like those based on the sector-based MULTUM,^22,257^ have also been made but struggle with the same technical challenges as conventional MR-TOFs. Moreover, ion images are increasingly distorted with the number of turns.^257^ The most recently published figures of merit of a MULTUM TOF using PEDA are a mass resolving power of 10,000 while maintaining a spatial resolving power of ∼1 μm.^22^ Furthermore, Verenchikov et al. proposed a further development of the aberration-corrected, ion-refocusing MR-TOF discussed above, which would be capable of stigmatic ion imaging, potentially without ion image distortions.^258,259^ This concept however, has not been verified experimentally yet. With the development of spatially-sensitive detectors with nanosecond resolution,^26,27,260^ it is likely that renewed interest will be given to stigmatic TOFs.

#### Fourier Transform (FT)-Based Hybrid Mass Analyzers

The highest mass resolving powers in MSI are achieved with Orbitrap and Fourier-transform ion cyclotron resonance (FT-ICR) mass analyzers,^261,262^ collectively termed FT-MS. Both mass spectrometers typically use quadrupole ion guides for mass range selection, eventual fragmentation, and transfer into ion traps. Ions are accelerated into a trapped orbital motion. A *m/z*-dependent ion frequency is determined in both instruments, the oscillating trapping motion in the Orbitrap and the rotational cyclotron motion in the FT-ICR. This frequency is then determined via Fourier transformation of the measured time-domain signal.^235^ Achievable mass resolving power in both Orbitrap and FT-ICR increases with field strength, respectively electric and magnetic, transient time, which is the time taken to measure the ions’ frequencies, and vacuum-dependent coherence time. Without custom modifications, Orbitrap mass analyzers can achieve mass resolving powers >1,000,000 at 200 *m/z*.^263^ High-end FT-ICRs can offer even higher mass resolving power, for instance MSI on a 21 T FT-ICR achieves a mass resolution of 1,600,000 at 400 *m/z* and <100 ppb mass accuracy.^264^ However, the large footprint of FT-ICRs, their complexity, and the need for a superconducting magnet, limit their widespread application in routine MSI.

The mass resolving power of FT-MS can be increased further using novel data acquisition systems, which enable longer transient times at the cost of imaging throughput and absorption mode. For instance, Kooijman et al. used the Booster (Spectroswiss, CH) on a 7 T FT-ICR to achieve a mass resolution beyond one million at 782 *m/z*.^233^ Likewise, using the Booster on a Q Exactive HF Orbitrap increased mass resolving power from 240,000 at 200 m/z to 1.4–1.5 million in the range of 700 to 900 m/z.^265^

A drawback of MSI with Orbitraps and FT-ICRs is low throughput as recording a high-resolution mass spectrum requires up to several seconds long transient times.^233^ Shorter transient times allow acquiring up to 40 mass spectra s^‑1^ on an Orbitrap, however only at a reduced mass resolution of 7,500 at 200 *m/z*.^266,267^ The acquisition rate of FT-ICRs and theoretically Orbitraps can essentially be doubled by accumulating ions in a separate ion trap while simultaneously analyzing another ion.^233^ In another approach Passarelli et al. built a SIMS instrument with a hybrid TOF/Orbitrap mass analyzer, in which the TOF is used for high throughput imaging, while the Orbitrap allows acquiring high resolution mass spectra at lower repetition rates.^164^

Further developments in FT MSI have been an increase in mass range by the introduction of ultra-high mass range (UHMR) Orbitraps,^268^ and the use of quadrupole notch filters to either selectively remove highly abundant ions, for instance MALDI matrix peaks, or to enrich lowly abundant ions in a subsequent ion trap. Given the finite charge capacity of ion traps, selected ion ejection improves sensitivity and enables visualizing low abundant species.^269^

#### Other Mass Analyzers

Quadrupole-based mass filters as standalone mass analyzers are rare in MSI as they only measure one *m/z* continuously and discard all other ions. To change the mass channel, the settings of the electric field of the quadrupole have to be changed. The scan rate at which the mass spectra are acquired is in the order of half a second for even a few thousand mass channels, which would lead to impractically long acquisition times, induce substantial damage to the sampled spot and thus alterations in the mass spectra.

Quadrupole trap, especially linear ion trap (LIT)-based instruments are occasionally reported as they are less expensive and more compact than most other analyzers. The sensitivity of LITs can effectively be enhanced by continuous ion accumulation, which is impractical for TOFs and FT-MS. LITs can also provide tandem mass spectrometry without much additional complexity.^270–272^ Most applications using quadrupole ion trap-based instruments have focused on quantifying metabolites and other molecules in lower *m/z* ranges.^272,273^

Magnetic sector based MSI instruments can, depending on their design, either measure a single *m/z* channel continuously, or 4-11 *m/z* channels simultaneously. The exact number of simultaneous mass channels depends on the number of detectors built into the instrument.^7,235^ Alternatively, the Wirtz lab introduced a focal plane detector consisting of a line of stacks of multichannel plate and delay line detectors, to record even more mass channels in parallel.^7^ The mass resolving power of multi-channel magnetic sectors can be as high as ∼15,000, however only by removing ions to reduce energy spread.^235^ Sectors for MSI are primarily used for elemental SIMS as they allow achieving the sensitivity necessary for highest spatial resolution imaging.^159^

### Ion Mobility Spectrometry (IMS)

Molecular MSI spectra can be complex, featuring many different, possibly isobaric and isomeric, ions at the same time. An additional separation step prior to ionization could simplify mass spectra, mitigate ion suppression, and increase the amount of identified compounds. However, chromatography-based techniques, are often too time-consuming to perform for every pixel in an image.^274^

A separation strategy that is suitable for MSI ion mobility spectrometry (IMS). IMS separates ions by their collision cross-section (CCS) with an inert gas, e.g. helium or nitrogen. While IMS does not alleviate ion suppression, IMS spectra can be recorded within the order of tens of milliseconds to a second with mobility-separated peaks that are a few milliseconds wide.^275–279^ Thus, IMS can be coupled to TOF MSI and provides advantages such as removing background ions, for instance MALDI matrix clusters.^277^ Additionally, given sufficiently different CCS values and high IMS resolving power, IMS can resolve structural isomers.^278^ As a result, when compared to MSI alone, IMS MSI can provide increased numbers of detected analyte peaks, potentially enhance identification of biomolecular compounds,^278,280^ and produce images with less chemical noise.^278^ Furthermore, CCS values provide an additional metric that can assist in peak identification and structure assignment. However, obtaining accurate CCS values from ion mobility data requires proper calibration, further improvements in repeatability, and the adaptation of IMS protocols to MSI.^275,281^ The disadvantages of hyphenating IMS to MSI are reduced throughput,^36,236,278^ increased ion losses especially for low-weight species due to radial ion diffusion,^277^ and increases in measurement file size and complexity as IMS adds another dimension to already large MSI data sets.^276^

Two IMS techniques, travelling wave ion mobility spectrometry (TWIMS) and trapped ion mobility spectrometry (TIMS), have predominantly been used in conjunction with MSI (Figure 8c and d). Other ion mobility techniques, such as field asymmetric waveform ion mobility spectrometry (FAIMS) or differential mobility analysis (DMA) have been less popular in MSI as they require discarding most ions.

TWIMS is similar to the conventional drift tube IMS (DTIMS) in that it consists of a tube in which a stationary gas counteracts an electric field pushing ions forward. Additionally in TWIMS, diffusion-driven ion losses are reduced by radially confining ions with alternating RF fields generated by stacked ring ion guides. Ions are propelled forward in a ‘surfing’ motion by periodic DC waves that travel through the TWIMS cell. With increasing CCS ions experience more collisions with the gas and ‘roll’ over more waves until they elute out of the TWIMS cell.^275,282^ Unlike in DTIMS, in TWIMS there is no simple mathematical relationship between drift time and CCS and a calibration is necessary to obtain ion mobility values.^283^

TWIMS MSI has mostly been used to remove chemical noise and as a rough gas-phase ion separation step.^219,276^ A capability to resolve isomers, e.g. lipid sn- or cis/trans-isomers, has to our knowledge not been demonstrated yet and a comparison of the resolving powers of different IMS techniques is difficult due to the use of different definitions and protocols.^275,281,284^ Advanced TWIMS instrumentation with longer flight paths has higher resolving power and may allow such isomer resolution. However, such advanced TWIMS comes at the cost of increased ion losses and drift times, which might make these less suited for MSI.^279,285^

In TIMS, ions are first accumulated in an ion trap while counter-directional gas flows through the TIMS cell, causing ions of different CCS to reach different equilibrium positions inside the trap.^286^ In the trap, radial RF fields reduce ion losses, while axial electric fields ensure that the gas flow cannot force ions out. This axial electric field is then gradually lowered so that ions with sufficiently high CCS and therefore collision-transferred momentum can escape the ion trap leaving ions with low CCS behind. Thereby, ions of different CCS are ejected over time.^286,287^ TIMS resolving power is tunable and proportional to the electric field gradient (EFG), with which the axial trapping potential is lowered. Unlike other IMS methods, resolving power is independent of TIMS cell length, allowing for space-efficient designs and their incorporation into, for instance, QTOFs.^236^ With high EFG scan times of 650 ms the resolving power of TIMS is sufficient to resolve some lipid sn- and cis-/trans-isomers given enough difference in CCS.^278^

### Tandem MS

Tandem MS or MS/MS is a technique to fragment molecular ions and produce a mass spectrum of their fragments. It is useful to distinguish compounds with identical or isobaric *m/z* and to help with molecular structure identification. MS/MS methods suited for MSI should ideally not impair throughput, have narrow precursor isolation windows, provide sufficient mass resolution to separate isobaric species, and in case of MALDI and SIMS, work well with single charged molecules.

The most prevalent MS/MS technique is collision induced, or activated, dissociation (CID or CAD, respectively) of a selected precursor ion. In CID, collisions with an inert background gas transfer energy into a molecular ion, which distributes this energy in its structure until the least stable chemical bond is broken, causing fragmentation and product ion information. CID is incorporated into many mass spectrometers, including imaging mass spectrometers.^36,267,288^ Usually, CID is performed in ion traps or quadrupoles in QTOFs, but TOF/TOFs, in which ions are pushed through a collision cell into another mass spectrometer, exist as well.^288,289^ The advantages of some TOF/TOFs are that all other ions stay on their trajectory, allowing MS/MS to be performed in parallel to normal imaging acquisition,^288^ and that multiple MS/MS measurements of precursor ions with sufficiently different *m/z* are possible within each initial TOF cycle.^289^ The disadvantages of TOF/TOFs are a larger footprint, higher collision energies leading to less predictable MS/MS spectra, broad precursor isolation windows of ∼4-10 Da,^288^ and that no precursor enrichment, as with ion traps, is possible.

Low energy CID is not capable of distinguishing all isomers, for instance it usually does not reveal the location of double bonds (db) in lipids, which is important for lipidomics. Possibilities to resolve double bonds in MSI are high-energy CID,^288^ on-tissue chemical derivatization,^290^ ion mobility with sufficiently high resolving power,^278,291^ ozone-induced dissociation (OzID),^292^ vacuum-ultraviolet photodissociation (UVPD), and electron impact dissociation (EID, sometimes referred to as electron-impact excitation of ions from organics (EIEIO)).

In OzID, ozone is leaked into an ion trap or ion mobility cell. Ozone reacts with unsaturated compounds, causing their selective fragmentation into a Criegee intermediate and a ketone (Scheme 1a).^290^ Only one of the products carries a positive net charge and is detected, while the other product is lost as a neutral. Since there are two possible reaction pathways, OzID MS/MS spectra show two characteristic peaks 16 Da apart from each other.

**Scheme 1:**
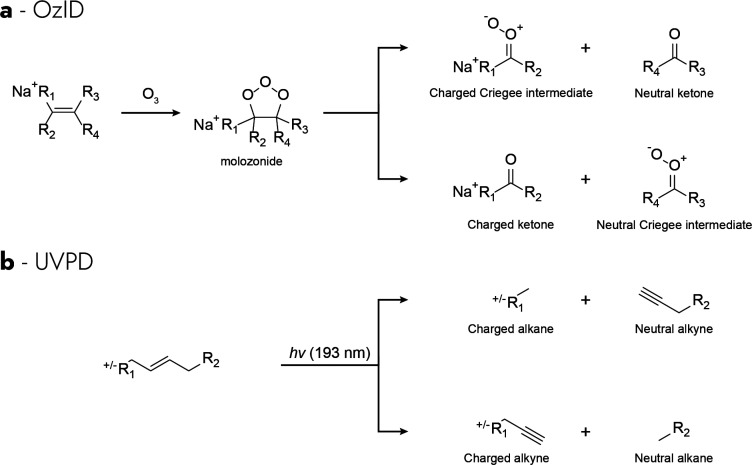
Double Bond Specific MS/MS Reactions^a^

a In OzID (a), charged alkenes undergo ozonolysis via 1,3-dipolar cycloaddition with ozone (O_3_). The resulting molozonide decomposes into a ketone and a Criegee intermediate. One of these products carries a charge and is detected with MS, while the other molecule is lost as a neutral. OzID MS/MS spectra therefore show two characteristic peaks 16 Da apart. UVPD (b) causes the cleavage of one of the C–C bonds adjacent to an alkene group. The products, of which only one is charged, are an alkane and an alkine with a mass difference of 24 Da (C_2_).

Furthermore, sequential or parallel CID/OzID fragmentation allows elucidating the esterification position (sn) of glycerophospholipids as well.^292,293^ An advantage of OzID over competing MS/MS methods is that it results in comparatively simple spectra with high yields of db-specific fragments allowing not only to identify but also to image less abundant lipids. The main drawback of OzID is a slow reaction speed. Ozonolysis is accelerated for positive polarity ions, by sodiating the sample prior to measurement, and by an increased ozone pressure in the reaction volume.^292,293^ The fastest image acquisition speed reported so far are 5 pixels s^‑1^.^293^

Alternatives to CID and OzID are UVPD and EID, which both are unselective fragmentation techniques and thus lead to information-rich, yet complicated MS/MS spectra.^294,295^ In UVPD irradiation with a UV laser beam, usually at 193 nm, causes ion fragmentation. UVPD can distinguish db-isomers as it causes the cleavage of one of the C-C bonds adjacent to the alkene bond, leading to the formation of an alkane and an alkyne (Scheme 1b). Only one of them is charged. MS/MS spectra therefore show two characteristic mass peaks 24 Da apart.^295^

In EID, ions are bombarded with electrons. The main difference to the more prominent electron capture dissociation (ECD) is the higher electron energy, which for lipids usually is in the range of 10-20 eV.^294,296,297^ Unlike ECD, EID is applicable to single charged ions, which are the dominant ion species for most MSI techniques. This applicability results from the fact that the electron bombardment does not lead to electron absorption by ions but merely to an energy transfer to the ions and subsequently their vibrational and electronic excitation.^298^ EID MS/MS spectra therefore show fragment ions similar to CID as well as homolytic bond cleavages. EID MS/MS can provide insights into the locations of double bonds and can distinguish trans-/cis-isomers in lipids.^294,296^

A disadvantage of both EID and UVPD is that only a small amount of the precursor ions fragment into characteristic ion pairs that yield information on functional groups, e.g. double bonds. For instance, UVPD of fatty acid anions yielded <0.1% diagnostic ion pairs.^299^ It therefore remains to be seen whether UVPD and EID will become more widely adopted for lipid isomer imaging.

In MSI, MS/MS spectra are often acquired automatically via data dependent acquisition (DDA). One strategy is to acquire a full mass spectrum in one pixel, and several MS/MS spectra on adjacent pixels.^300^ In practice, this limits spatial resolving power and resolution as only the pixels used for acquiring full spectra are used to construct images. This issue can be mitigated by oversampling at the expense of increased acquisition time.^301^ A partial solution to the increased acquisition time of DDA MSI was presented by Ellis et al., who performed MS/MS in a linear ion trap (LIT) while acquiring mass spectra of an adjacent sample position on an Orbitrap.^302^ The advantages of this parallel acquisition method is that throughput is not impaired by doing MS/MS in addition to MSI, and that LITs are more sensitive than Orbitraps. Another approach that combines trapped ion mobility spectrometry (TIMS) with MS/MS, entails the acquisition of an image in full MS mode followed by measuring MS/MS spectra of selected precursors on unused sample spots distributed over the sample.^303^ The advantage of using TIMS is that multiple precursors can be enriched in a first ion trap, separated by their ion mobility in a second ion trap, and then fragmented and analyzed in a mass spectrometer. This technique is known as parallel accumulation – serial fragmentation (PASEF).^254^

### Imaging Throughput

We propose to define imaging throughput as the average sampling speed *S* (equivalent to pixels s^‑1^) of an MSI image:

9 $$S=\frac{A_{image}}{spatial\;resolution^{2}\cdot t_{total}}$$

Where *A*_*image*_ is the image area, *spatial resolution* is equal to pixel size (assuming a square pixel), and *t*_*total*_ is the image acquisition time.

Compared to most other imaging techniques, MSI has, even without considering the times needed for sample preparation and data analysis, been a technique with low throughput. Low throughput increasingly presents a bottleneck in MSI as new applications require ever higher spatial resolving power.^304–306^ Following eq ([Disp-formula eq8]), linear improvements in spatial resolution cause quadratic increases in the amount of image pixels to be scanned, leading to prolonged acquisition times in microprobe-mode MSI. Consequently, the applicability of high spatial-resolution MSI for large-cohort studies or time-sensitive applications, for instance in a clinical environment,^11,307^ will be limited unless imaging throughput also increases non-linearly.

In this section we review advancements in imaging throughput. We do not discuss multimodal approaches as these technically do not lead to higher throughput in MSI but only “outsource” imaging throughput to another imaging technique. Furthermore, we will not discuss needed increases in sensitivity to increase imaging throughput as this topic has already been discussed in other sections.

Speedups in imaging throughput have been achieved by the use of lasers or ion beams with higher repetition rate or even continuous operation (i) to reduce the time needed for ionization.^101^ Ion source washout and transfer times to the mass spectrometer (ii) are negligible for most ionization techniques except for ambient laser ablation-based MSI, in which these times have been reduced for LA-ICP MSI by a combination of optimized laser, carrier gas, ablation cell and transfer pipe properties. As a result LA-ICP TOF MSI with one laser shot per pixel reaches sampling speeds of several 100 pixels s^‑1^.^127,135^ The time needed to move between pixels (iii) can be reduced by continuous rather than stepwise sample motion to avoid acceleration and deceleration periods.^8,308^ Continuous sample stage motion however, is not as precise for small pixel sizes as stepwise motion.^309^ Furthermore, when surfaces are depleted rapidly, ionizing at high or continuous repetition rate would not be beneficial unless the sample is moved correspondingly quickly, causing high wear on the stage motors.^308,309^ An alternative with even faster imaging throughput than continuous sample movement is to move the laser or ion beam instead of the sample. In SIMS, ion beam rastering is done routinely with the help of deflector fields while moving the sample in discrete steps to cover larger areas in so-called “mosaic imaging mode” achieving acquisition rates of up to several 100 pixels s^‑1^ on TOF and sector instruments.^8,237^ In MALDI, laser beam movement is achieved by the use of two motorized mirrors prior to laser beam expansion and focusing.^67,309,310^ Following proper optical and ion optical realignment, fast mirror movement combined with slow sample stage motion, allow for an acquisition speed up to ∼40–50 pixels s^‑1^ and uniform sample irradiation on commercially available microprobe-mode MALDI MSI instruments.^67,309,311^

Last, the time needed for measuring a complete mass spectrum (iv) is shortest with TOF mass spectrometers. Although the spectral acquisition rate of FT-MS analyzers has been improved by the use of stronger electromagnetic fields, sparse sampling,^312^ and parallel data accumulation and analysis,^233^ FT-MS imaging throughput is still an order of magnitude slower than microprobe-mode TOF MSI.

TOF mass analysis time can be decreased either by restricting high-end mass range or by accelerating ions to higher speeds. Higher speeds can improve mass resolving power and mass analysis time. However, the degree to which ions can be accelerated is limited by electronic safety considerations, voltage and timing reproducibility, and ultimately by the onset of relativistic effects. Thus, to increase imaging throughput of TOF MSI further other approaches are needed. Multiplexing TOF spectra acquisition in time, for instance via Hadamard transformation or EFP,^241,313^ is challenging as MSI spectra are often complex, and so far only minor improvements in the imaging throughput of MR-TOFs have been achieved using EFP.^249^

Another promising alternative is multiplexing in space via fast mass microscopy. In a mass microscope entire ion images are separated by mass and projected onto a spatially-sensitive pixelated detector with nanosecond resolution.^8^ On the event-based detector, for instance a Timepix3 or 4,^26,27^ each pixel records its own mass spectrum, thus allowing for the simultaneous acquisition of tens of thousands of mass spectra.^8,23^ Larger images can be constructed from individual images that were recorded while continuously moving the sample.^8^ Spatial resolving power in mass microscopy is independent of the ionizing beam’s spot size. Thus, fast mass microscopy allows for simultaneously high spatial resolving power and at least 3-4 orders of magnitude higher throughput than microprobe-mode MSI.^8^ Notably, even higher acquisition rates seem possible by increases in fov, detector size, sensitivity, and by the points (i) and (iii) discussed above. The main disadvantages of fast mass microscopy are poor mass resolving power compared to microprobe-mode MSI, and that it can only performed under UHV. The UHV requirement removes possibilities for coupling mass microscopy to techniques requiring EP or AP, such as MALDI-2, IMS, and MS/MS, and restricts its applicability to SIMS, UHV MALDI, and other methods not discussed here, such as laser desorption ionization (LDI).

## Targeted MSI

### Immunohistochemistry (IHC)

It is sometimes difficult to perform untargeted MSI of proteins and RNA at high spatial resolutions due to low abundance, the presence of ion suppression effects and the difficulty to unambiguously identify proteins, even at high mass resolution.^314^ Spatial proteomics and transcriptomics experiments have therefore more routinely been done with immunofluorescence microscopy (IFM).^3^ In IFM, the sample is stained with antibodies to which a fluorophore group is attached. Emitted light from these fluorophore groups allows localizing proteins in tissues. IFM is highly sensitive, usually less expensive per image than MSI, can be noninvasive,^315^ and has a spatial resolving power of ≈200-350 nm for diffraction-limited light microscopy. However, IFM suffers from two drawbacks: noise caused by autofluorescence,^3,125^ and spectral overlap of broad absorption and emission bands, which makes it difficult to image more than 3-5 antigens at a time and therefore to study protein-protein interactions.^1–3,316^ Strategies to overcome the issue of spectral overlap are either based on cycling consecutive staining and bleaching steps, which however lead to extended acquisition times and might be detrimental for the integrity of the sample,^2,317^ or on barcoding,^318^ which requires a sophisticated and error-robust experimental design.

MSI, on the other hand, suffers less from common noise sources that are problematic to IFM and is capable of simultaneously acquiring a practically unlimited amount of mass channels. Bandura et al. realized that the disadvantages of fluorescence based techniques could be bypassed by using antibodies, with a mass tag instead of a fluorophore tag, and a mass spectrometer instead of an optical detector.^319^ This new technique, termed mass cytometry (MC), was first introduced as alternative to flow cytometry,^319^ and later adapted to LA-ICP MSI as imaging mass cytometry (IMC),^125^ to SIMS as multiplexed ion beam imaging (MIBI),^320^ and to MALDI and DESI immunohistochemistry (IHC) MSI.^321,322^

The most common mass tags are rare earth metals, e.g. lanthanides (La),^319^ as they are lowly- to nonabundant in biological tissue, and can easily be detected with LA-ICP (Figure 9) and SIMS.^125,320^

**Figure 9:**
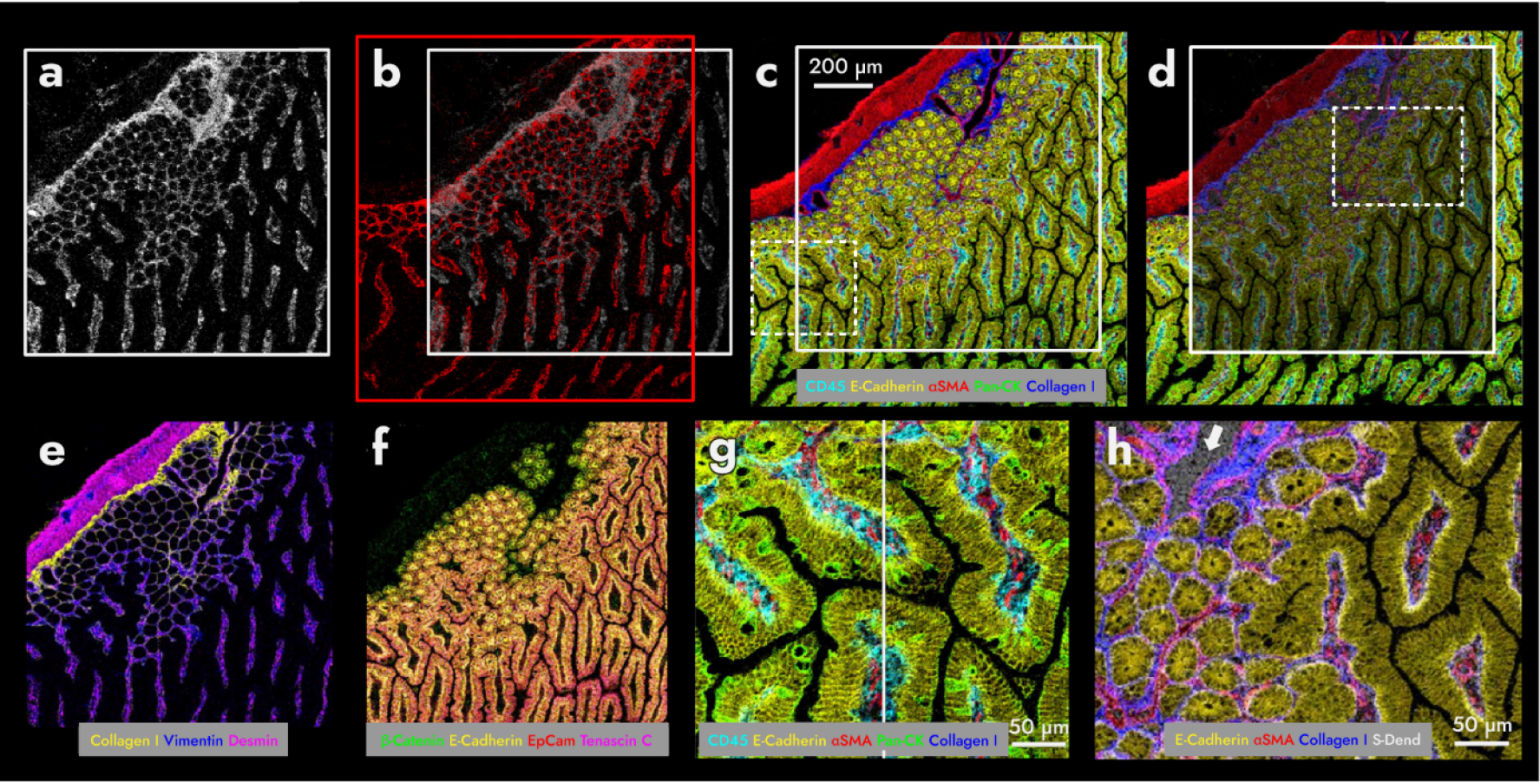
An example of targeted IHC MSI – LA-ICP MSI of mouse duodenum of an in-vitro injected ^159^Tb-marked polysarcosine-modified dendrimer drug-delivery system (S-Dend) followed by immunostaining and imaging mass cytometry (IMC) of 24 proteins (adapted with permission from Strittmatter, N.; England, R. M.; Race, A. M.; Sutton, D.; Moss, J. I.; Maglennon, G.; Ling, S.; Wong, E.; Rose, J.; Purvis, I.; Macdonald, R.; Barry, S. T.; Ashford, M. B.; Goodwin, R. J. A. Method to Investigate the Distribution of Water-Soluble Drug-Delivery Systems in Fresh Frozen Tissues Using Imaging Mass Cytometry. *Anal. Chem*. **2021**, *93* (8), 3742–3749. (ref 323). Copyright 2021 American Chemical Society.). Since S-Dend is water soluble and washed away by immunostaining, Strittmatter et al. first performed LA-ICP MSI of S-Dend (a; (b) is an overlay of (a) with the mass image of an adjacent tissue section), and then IMC on the same tissue section (c–h). In (c) the region previously ablated to measure S-Dend is highlighted by a white box. In (d), (a) is overlaid onto (c) with 50% opacity. The images (e) and (f) are of connective tissue and mucosa substructures, while (g) and (h) are zoom-ins into the areas marked with dashed boxes in (c) and (d), respectively. A white vertical line in (g) separates previously ablated (right) from nonablated tissue (left). The white arrow in (h) highlights a large blood vessel. In (c), (d), (g), and (h) the colors cyan, yellow, red, green, blue, and white correspond to CD45, E-cadherin, αSMA, pan-CK, collagen I, and S-Dend, respectively. In (e), yellow, blue, and magenta represent collagen I, vimentin, and desmin, while in (f) green stands for β-catenin, yellow for E-cadherin, red for EpCam, and magenta for tenascin C, respectively. The scale bars are 200 (a–f) and 50 μm (g and h), respectively.

La-labeled antibodies are available for both proteins and mRNA,^324^ and their detection efficiencies have been optimized by increasing the amount of metal per antibody, especially by the use of immune-SABER.^325^ La-imaging is either done with LA-ICP MSI, known as imaging mass cytometry (IMC) or with SIMS known as multiplexed ion beam imaging (MIBI).^125,320^ These techniques have already proven to be valuable in biomedical research as they allow studying the spatial distributions and interactions of theoretically >100 biomarkers simultaneously. In practice, the current multiplexing of lanthanide tags is ∼40 tags on a single tissue.^125,316^

Another immunolabeling approach uses synthetic peptides connected to the antibody via a photocleavable molecular linker. After the antibody staining, the sample is irradiated with a UV lamp. Irradiation cleaves the bond and frees the peptide tags. Following matrix application, these peptides can then be detected with MALDI.^314,321^ Such synthetic peptide-based mass tags offer even higher possibilities for multiplexing than the isotopes of heavy metals, as they can be engineered to thousands or even tens of thousands of mass channels. An alternative concept was proposed for DESI, in which antibodies are labeled with boronic acidic mass tags. These tags undergo acidic cleavage and protonation in secondary DESI droplets, allowing their detection with mass spectrometry.^322^

When comparing these three approaches, IMC has the highest ionization efficiency, as the hot plasma ionizes most metals with >90% efficiency.^124^ Ion suppression does not occur but the sample is destroyed during laser ablation. State-of-the-art commercial IMC instruments achieve an acquisition speed of up to 400 pixels s^‑1^, meaning that at highest spatial resolution of 1 μm it takes ∼42 min to image 1 mm^2^.

MIBI is less destructive and has the highest spatial resolving power (<50 nm) of all IHC-based MSI methods.^326^ However, MIBI also has low ionization efficiency and might be subject to ion suppression. MIBI requires high sensitivity to probe nanoscale volumes. It is therefore advisable to use bright, reactive, DC mode ion guns and mass spectrometers with high ion transmission.^237,320^ Magnetic sector instruments are useful in that regard but have a limited number of mass channels, making TOFs more attractive for MIBI.^237^ Low sensitivity in combination with the limitations of microprobe-mode MSI leads to comparatively low throughput in MIBI, despite being able to scan several 100 pixels s^‑1^.^237^

In comparison to MIBI and IMC, microprobe-mode MALDI- and DESI-IHC have worse spatial resolving powers of ∼1–5 μm and ∼50 μm respectively,^227,314^ scan only at up to ∼50 pixels s^‑1^ and 1–5 pixels s^‑1^ respectively,^67^ and are prone to ion suppression effects, which can however be mitigated, e.g. by adding a sterically protected, charged side group to the peptides (see chapter on OTCD). However, MALDI and DESI will allow for a higher number of labels and for combining untargeted metabolomics with targeted proteomics using the same technique.^314,321^ This is also to some extent possible with SIMS but requires using a different ion gun and has harder ionization and a smaller mass range than MALDI.^217^ Furthermore, improvements in throughput in both MALDI and MIBI seem possible by the use of mass microscopy.^8^ Additionally, expansion microscopy MSI combined with further sensitivity enhancements could boost the effective spatial resolving power of every IHC MSI method.^17,18,325^

### Stable Isotope Labeling (SIL)

Another targeted MSI approach is to administer stable isotopic labels (SIL), for instance an amino acid containing ^13^C or ^15^N, to one or several living organisms via nutrition or infusion. The organisms are sacrificed at different time points and their tissue is analyzed. By measuring the spatial distribution of the isotopic ratios of the labeled standard and its metabolites, one obtains images that show the uptake and the metabolism of the standard over time. In contrast to other techniques commonly used to monitor metabolite uptake, such as radioautography and positron emission tomography (PET), no radioactive isotopes are required.

SIL MSI was first introduced to high resolution elemental SIMS under the term Multi-isotope imaging mass spectrometry (MIMS),^327,328^ and was later adopted to MALDI MSI.^329–332^ SIL-MALDI MSI can benefit from an additional OTCD step as many metabolites, for instance amines, can be suppressed by other molecules.^329^

### Quantitation

Quantitative MSI (Q-MSI) of biologically relevant analytes from tissues has large potential for many biomedical applications. However, Q-MSI has been challenging to perform for all MSI ionization methods due to the inhomogeneity of tissues leading to different analyte extraction, desorption, and ionization efficiency for each pixel.^333,334^ Furthermore in case of MALDI and ME-SIMS, matrix coverage may also vary. Post-processing normalization techniques, such as total ion count (TIC) normalization, can partially compensate for signal fluctuations between pixels, but not to the degree necessary for absolute quantitation.^333,335^ Thus, every pixel should be normalized with an internal standard sprayed evenly under or on top of a tissue.^272,273,336–338^ As both analyte and internal standard should behave chemically identically but differ in mass, internal standards are often a deuterated or ^13^C labeled version of the analyte.^272,339^ Precision and accuracy of Q-MSI increase when multiple internal standards at different concentrations are used as they allow obtaining a calibration curve for every pixel.^339^ Furthermore, it is advisable to use MS/MS to separate isobaric ions.^340^ The disadvantages of using isotopically labeled standards are high cost, additional sample preparation steps that might cause sample delocalization, and that quantitation is limited to one molecule. Furthermore, Q-MSI results cannot always be directly compared with bulk methods, for instance, laser capture microdissection followed by tissue homogenization and LC-MS,^339^ as MSI, particularly static SIMS, probes a surface not a volume.

## Multimodal Imaging

### Definition and General Considerations

We propose to define multimodal imaging as the advantageous combination of multiple complementary imaging or ionization techniques. The aim of multimodal imaging in MSI is to benefit from its strengths while compensating its remaining weaknesses compared to other techniques. Such compensated weaknesses include low imaging throughput, low spatial resolving power, and ion suppression.

Multimodal imaging holds great promise but also bears several challenges. First, the information gained by the two modalities should be comparable. For instance, it is difficult to directly correlate Raman bands with single MSI peaks as MSI has a much larger specificity and sensitivity than Raman.^341^ Another example is the combination of SIMS, a surface probing method, with transmission electron microscopy (TEM), which also shows subsurface features.^342^ Second, sample preparation and acquisition workflows should be compatible and not lead to artefacts in the other modality. For that reason, imaging with destructive methods, as most MSI techniques are, should be performed last. An exception are imaging techniques based on dye or immunostaining where the sample preparation causes the removal or delocalization of metabolites and might even give rise to additional mass peaks. Furthermore, many MSI techniques require sample dehydration and are performed under vacuum, which can cause sample shrinking and fractures. Even more difficult is the coupling of MSI with in vivo methods, such as magnetic resonance imaging (MRI),^343^ where artefacts can be induced during all steps of sample preparation.

Third, as multimodal imaging is often performed on different instruments, the resulting images must be aligned via so-called image registration. Good image registration must overcome differences between the images, including position, orientation, pixel size, artefacts, and image features. For an in-depth review on image registration for MSI we recommend an article by Balluff et al.^344^ Briefly, coarse image registration can be performed manually by the user, who identifies several landmark regions in both images followed by an algorithm performing the necessary image transformations. A standard manual workflow for image registration between MALDI MSI and optical microscopy is to record optical images before and after performing MSI and eventual staining, and then to first co-register these images before co-registering the MSI image with the first image.^344^ This approach works well for images with low spatial resolution, but needs an increased number of fiducial markers surrounding the sample at higher spatial resolutions. For instance, image registration with submicron precision may require up to 3,000 markers.^345^ These markers would have to be added manually or originate from the sample, for instance Patterson et al. suggested using laser burn marks to align MALDI with autofluorescence images.^346^ Still, automated image registration methods will be needed for the actual image registration. Automated image registration, however, has not become routine yet, as it can struggle with correctly recognizing features in different modalities, for instance due to different pixel sizes, noise, strongly different, or too many similar features.^344^

Thus, a better alternative to image registration, particularly for high-spatial resolution MSI, is to perform all imaging on the same instrument, which would also be cheaper than two separate instruments, but so far only a few prototypes have been developed, mostly for combining TOF-SIMS with MALDI,^240,347,348^ and magnetic sector SIMS with SEM,^150^ TEM,^342^ and helium ion microscopy (HIM).^185,203^

### Selected Examples

In this subsection, we present the most promising strategies for multimodal MSI. We exclude rare approaches, such as MSI combined with atomic force microscopy (AFM),^203^ vibrational spectroscopy,^341^ MRI,^343^ and spatial transcriptomics.^349^ We also do not discuss guided MSI approaches that image the surface with a fast imaging technique followed by slower MSI of selected locations as such approaches, by themselves, improve region selection but do not increase imaging throughput.

Tissues are often analyzed with hematoxylin & eosin (H&E) stained light microscopy, arguably a “gold standard” in pathology.^350–352^ H&E is a low-cost method to visualize cell nuclei, extracellular matrix, and cytoplasm at a spatial resolving power of ∼200-350 nm. Furthermore, the rise of label-free virtual staining might increase the prevalence of stained light microscopy images in pathology even further.^353^ However H&E stained light microscopy lacks detailed chemical information making it complementary to molecular MSI. Ščupáková et al. presented a method that based on the distance between cell nuclei first estimates the boundaries of single cells in tissue, and then uses the findings to segment MALDI MSI data of issue into single cells.^354^ This in situ approach allows correlating single cell metabolomics with their environment, while most other studies are performed ex situ on dried single cells dispersed on a slide.^160,345^ In situ single cell MSI has shown great potential for both fundamental lipidomics,^355^ as well as improved clinical diagnostics.^306^

Two other techniques well-suited for multimodal MSI are fluorescence microscopy and SIMS coupled to MALDI MSI. While MSI increasingly gains the capability to image proteins and RNA via targeted IHC approaches, fluorescence allows mapping selected biomolecules at higher spatial resolution than currently achievable with MSI. For example, Geier et al. studied mussel tissue with AP MALDI followed by localizing two symbiotic gammaproteobacteria with fluorescence in-situ hybridization (FISH, Figure 10).^315^

**Figure 10:**
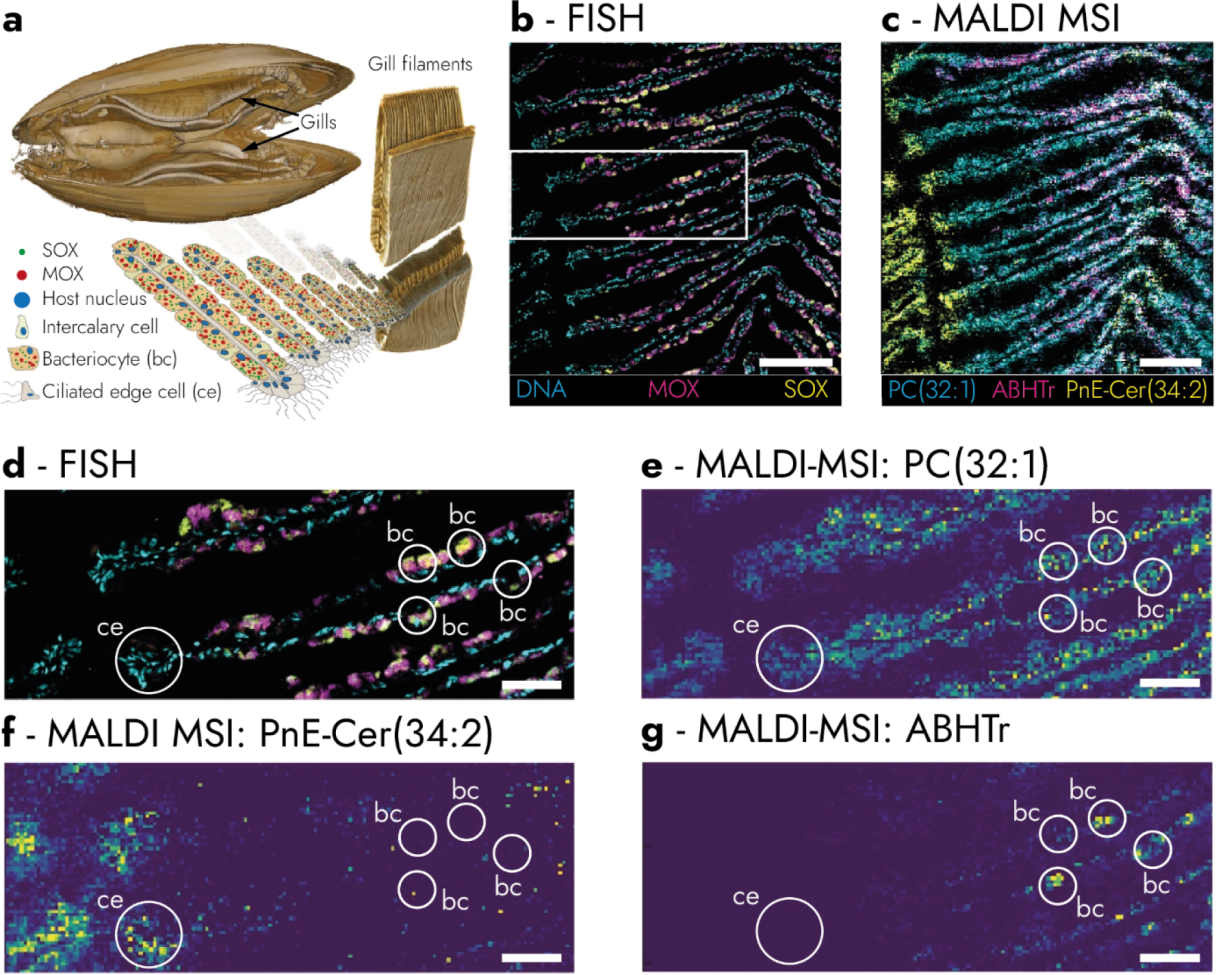
AP MALDI MSI and fluorescence in situ hybridation (FISH) imaging of gill filament tissue sections of a Bathymodiolus deep-sea mussel (adapted with permission from Macmillan Publishers Ltd: NATURE MICROBIOLOGY, Geier, B.; Sogin, E. M.; Michellod, D.; Janda, M.; Kompauer, M.; Spengler, B.; Dubilier, N.; Liebeke, M. *Nat. Microbiol.* **2020**, *5* (3), 498–510 (ref 315). Copyright 2020.). A micro-computed tomography model (a) depicts the location of the gills. Parts of the gill tissue, the bacteriocytes (bc), are co-populated by sulfur and methane oxidizing symbionts (SOX and MOX, respectively), while the ciliated edge (ce) consists only of host cells. The symbionts and their host were imaged with FISH (b). The colors cyan, magenta, and yellow correspond to the host DNA, MOX, and SOX, respectively. In (c), the spatial distributions of phosphatidylcholine PC(32:1), 35-aminobacteriophane-32,33,34-triol (ABHTr), and phosphonethanolamine ceramide PnE-Cer(34:2), were measured with AP MALDI MSI at 3 μm pixel size. Cyan, magenta, and yellow correspond to PC(32:1), ABHTr, and PnE-Cer(34:2), respectively. A white box in (b) highlights the region chosen for normalized zoom-ins (d–g). ABHTr can only be observed in the bacteriocytes whereas PnE-Cer(34:2) is solely found in symbiont-free host tissue. This multimodal approach allowed the authors to map chemical interactions between host and symbiont. The scale bars correspond to 150 (b and c) and 50 μm (d–g), respectively.

The combination yielded insight into the metabolic interactions between a host and its symbionts. SIMS and MALDI have been combined in one instrument with shared vacuum system and mass analyzer several times.^240,347,356^ SIMS is complementary to MALDI as it offers higher spatial resolving power with both SIMS and secondary electron detection,^240^ and provides access to elements and low-mass metabolites.

Image fusion can be used to overlay and merge the chemical information of MSI images with images recorded with a higher resolution technique, for instance light microscopy,^357^ or EM to predict how MSI images would look like at higher spatial resolving power.^342,357,358^ Image fusion was originally developed for satellite imagery.^342^ Techniques to split images into these components range from saving MSI images first as RGB and then as HSV image,^342,359^ to more sophisticated techniques like principle component analysis (PCA) and non-negative matrix factorization (NMF).^357,358,360^ Image fusion is only possible, if both images contain the same spatial features, which can be found via automated image correlation analysis.^357,358^ Prior to image fusion, both images need to be accurately co-registered. Furthermore, data processing steps, such as normalization, baseline correction, as well as noise and artefact removal, are helpful as well.^358^ The resulting fused images help visually in correlating mass information to structure not visible with MSI, for instance (sub-)­cellular structures. However, care must be taken concerning artefacts,^342,358^ and the images to be fused should always be inspected individually as well.

## Selected Application Examples

MSI is highly versatile and therefore has numerous applications in material,^7,361^ earth,^362^ and life sciences.^3,4,9,11,48,149,159,305^ Here, we highlight some examples of MSI applied to cancer diagnostics, fundamental single-cell research, and drug development.

### Cancer Diagnostics

Most, if not all, diseases are caused by or display a biochemical dysfunction detectable with mass spectrometry. This applies in particular to cancer,^11,363^ where combined genetic mutations, elevated cell proliferation, and the Warburg effect result in strong differences in protein and metabolite expression compared to healthy tissue. MSI is well-suited to study tumors in tissues, and indeed studies on lung and pancreatic cancer have demonstrated that untargeted MALDI and DESI MSI can distinguish tumor from healthy tissue with accuracies exceeding 98%.^364,365^ This high classification accuracy outperforms the current “gold standard” method used in pathology, H&E stained light microscopy, which exclusively relies on morphological features, which in some cases may not be specific to cancer. Furthermore, H&E image analysis requires the subjective image analysis by a trained pathologist, and different pathologists can arrive at different conclusions.^350,352^ Thus, patients are often misdiagnosed, necessitating additional treatment.^366^ If MSI ever achieves the throughput and spatial resolving power simultaneously needed for its application in a clinical setting,^11,307^ it could become a standard tool for pathologists that is complementary to H&E imaging of cryosections. In addition, untargeted MSI can yield further valuable information, such as metastatic progression,^367^ cancer subtype,^306^ statistically predicted patient outcome,^368^ or therapy response.^369^

A main challenge in cancer treatment is tumor heterogeneity, which is why many researchers believe that personalized diagnostics and treatment plans will be more effective than a “one-size-fits- all” cancer drug.^3,370,371^ Tumors represent complex ecosystems consisting not only of cancer cells, but also of various stroma cells, like fibroblasts or immune cells, blood vessels, and potentially altered extracellular matrix. Within this ecosystem, cancer cells interact with their tumor microenvironment (TME) causing other cells to aid them with nutrient supply, cell proliferation, protection against cancer drugs, and metastasis.^370,371^ Developing a holistic picture of TMEs requires not only mapping the spatial distributions of metabolites but also of RNA and proteins.^3^ Untargeted single-cell MSI of proteins is difficult due to their low abundance and ion suppression, but this issue can be bypassed with targeted multiplexed techniques, such as IMC,^3,125^ MIBI,^320^ and MALDI-IHC.^314^ For instance Jackson et al. used IMC and a panel of 35 antibody markers map breast cancer tissue from 352 patients.^316^ The obtained data revealed new subtypes of breast cancer, which could be associated to patient survival rate, genomic features, and treatment response.^316,372^ This work thus paves the way towards making personalized treatment recommendations. Similar work was conducted by Sorin et al. on lung cancer.^373^ Meanwhile, Keren et al. used MIBI to investigate triple negative breast cancer and found tumor subtypes based on whether and how immune cells have entered the tumor. Tumors with distinct immune cell compartments were associated with higher patient survival compared to tumors without immune cell infiltration and tumors with mixed cancer and immune cells.^374^ Another MIBI study on the TME of ductal carcinoma in situ (DCIS) showed that patients with a thinner myoepithelium layer correlated with a more reactive desmoplastic stroma are less likely to develop invasive breast cancer.^375^

### Fundamental Single-Cell Research

With ever higher spatial resolving power and sensitivity as well as with the development of multimodal techniques, MSI is becoming a viable tool in the analysis of single cells and their substructures.^9,376^ Early work used high-resolution SIMS of elements and stable isotope labels to map their uptake into the cell metabolism. Steinhauser et al. used this approach by incorporating ^15^N labeled thymidine into gestated mice to investigate the immortal DNA stand hypothesis.^327^ Four weeks after birth, all excess ^15^N in small intestinal crypt cells had depleted, thus contradicting the immortal DNA strand hypothesis.

In more recent SIMS works, Pareek et al. used GCIBs to show that de novo purine synthesis in HeLa cells is highly localized and limited to a few “hotspots” near the mitochondria.^377^ This and other findings established that de novo purine synthesis occurs in multienzyme complexes rather than in several delocalized, less efficient enyzmes.^377^

High spatial-resolution MALDI MSI is also increasingly being applied to single cell research.^9^ An example for a fundamental single-cell MALDI study is that Capolupo et al. showed that dermal fibroblast subtypes not only exhibit a different lipid profile, but also that sphingolipids control fibroblast heterogeneity.^355^

### Drug Development

Developing novel medication is a tedious and expensive process with high failure rate. MSI, DESI and MALDI in particular, can facilitate several stages of this process from target discovery to legally mandatory absorption, distribution, metabolism, and excretion (ADME) studies.^378,379^ For instance, Strittmatter et al. used a multimodal approach to verify that the drug gemcitabine preferentially accumulates and metabolizes at its target, pancreatic dual adenocarcinoma tumor cells.^380^ In another example, Shariatgorji et al. used DESI and MALDI MSI combined with OTCD to study the effect and metabolism of 6-hydroxydopamine, a drug that induces Parkinsonism, L-DOPA, a drug that counteracts Parkinson’s disease, and other psychoactive substances, like amphetamine, on the spatial distributions of neurotransmitters in rat brain.^381^ An issue with administering L-DOPA to Parkinson patients is that its long-term use can cause additional complications, like L-DOPA-induced dyskinesia. A follow-up MSI study by Fridjonsdottir et al. on monkey brains found increased L-DOPA levels in the brains of monkeys with dyskinesia, pointing towards a dysregulated L-DOPA metabolism.^382^ In another study investigating drug excretion and off target effects, van Assche et al. developed a protocol to perform quantitative MSI to study the uptake of doxorubicin, a drug known to cause heart disease, into mouse heart and kidney.^383^ In another instance, Nilsson et al. used MALDI MSI to show that the antibiotics colistin and polymyxin B accumulate in the renal cortical regions of the kidney, which might help explain their nephrotoxicity and could in future help to develop safer antibiotics.^384^

## Concluding Remarks

MSI has entered the mainstream of common analysis methods with a wide and increasing range of application areas from material and earth to life sciences, most notably cancer and ADME studies.

This rise in popularity was preceded by numerous technological advancements over the last two decades. MALDI MSI has turned into a versatile tool for in situ single cell analysis following the developments of MALDI-2, <1 μm and 5 μm resolving powers in transmission and reflection mode, and due to innovations in uniform matrix deposition and OTCD. Complementary to MALDI, LA-ICP MSI has become capable of mapping elemental distributions in tissues at 1 μm spatial resolving power and acquisition speeds of several 100 pixels s^‑1^ following the construction of novel ablation cells. Molecular SIMS has evolved with the introduction of GCIBs and of instruments with increased cryogenic capabilities. Meanwhile, elemental SIMS has profited from the development of novel monatomic ion guns and its increasing combination with SEM and HIM. DESI now routinely achieves a spatial resolving power of 50 μm thanks to the introduction of stiffer, more rigidly positioned sprayers.

Furthermore, MSI has benefitted from the continuing development of better instrumentation from higher mass resolution TOF and FT-MS analyzers, improved MS/MS capabilities, and the addition of ion mobility spectrometry. The combination of these technologies can greatly aid with molecular identification and structural analysis. Imaging throughput has been optimized as well, particularly for MALDI MSI, but seems limited in microprobe-mode. Here, the development of faster pixel-based detectors could lead to a renaissance of mass microscopy as a high throughput and simultaneously high spatial resolving power technique. Last, the introduction of targeted, highly multiplexed MSI of proteins bypasses the difficulties of untargeted intact protein identification. Targeted MSI now allows studying in detail the interactions of proteins in tissues, particularly in tumor microenvironments where already several breakthroughs have been achieved using IMC and MIBI. Moreover, together with untargeted MSI of metabolites and multimodal approaches, targeted MSI paves the way towards an ever more holistic understanding of the local chemistry in tissues.

Achieving such understanding, however, requires further improvements in instrumentation and method development, especially in sensitivity and spatial resolving power. Additionally, untargeted MSI needs more accurate molecular identification. Isomers in particular must be resolvable as many structurally different metabolite and lipid isomers may have different biological functions. The need for ever more capable MSI instruments will likely be accompanied by a demand for cheaper, easy-to-use, high throughput MSI instruments for large cohort studies and routine clinical diagnostics. Last, effective multimodal MSI requires development of more efficient image registration algorithms or improved multimodal MSI instruments. Such improvements will enable MSI to contribute towards obtaining a holistic understanding of spatial biology of single cells in tissue.
